# Pediatric Drug Development: Reviewing Challenges and Opportunities by Tracking Innovative Therapies

**DOI:** 10.3390/pharmaceutics15102431

**Published:** 2023-10-06

**Authors:** Cátia Domingues, Ivana Jarak, Francisco Veiga, Marília Dourado, Ana Figueiras

**Affiliations:** 1 Univ Coimbra, Laboratory of Drug Development and Technologies, Faculty of Pharmacy, 3000-548 Coimbra, Portugal; cdomingues@ff.uc.pt (C.D.); jarak.ivana@gmail.com (I.J.); fveiga@ci.uc.pt (F.V.); 2 LAQV-REQUIMTE, Laboratory of Drug Development and Technologies, Faculty of Pharmacy, University of Coimbra, 3000-548 Coimbra, Portugal; 3 Univ Coimbra, Institute for Clinical and Biomedical Research (iCBR) Area of Environment Genetics and Oncobiology (CIMAGO), Faculty of Medicine, 3000-548 Coimbra, Portugal; mdourado@fmed.uc.pt; 4 Institute for Health Research and Innovation (i3s), University of Porto, 4200-135 Porto, Portugal; 5 Univ Coimbra, Center for Health Studies and Research of the University of Coimbra (CEISUC), Faculty of Medicine, 3000-548 Coimbra, Portugal; 6 Univ Coimbra, Center for Studies and Development of Continuous and Palliative Care (CEDCCP), Faculty of Medicine, 3000-548 Coimbra, Portugal

**Keywords:** pediatrics, nanoparticles, gene therapy, cell- and tissue-based therapy

## Abstract

The paradigm of pediatric drug development has been evolving in a “carrot-and-stick”-based tactic to address population-specific issues. However, the off-label prescription of adult medicines to pediatric patients remains a feature of clinical practice, which may compromise the age-appropriate evaluation of treatments. Therefore, the United States and the European Pediatric Formulation Initiative have recommended applying nanotechnology-based delivery systems to tackle some of these challenges, particularly applying inorganic, polymeric, and lipid-based nanoparticles. Connected with these, advanced therapy medicinal products (ATMPs) have also been highlighted, with optimistic perspectives for the pediatric population. Despite the results achieved using these innovative therapies, a workforce that congregates pediatric patients and/or caregivers, healthcare stakeholders, drug developers, and physicians continues to be of utmost relevance to promote standardized guidelines for pediatric drug development, enabling a fast lab-to-clinical translation. Therefore, taking into consideration the significance of this topic, this work aims to compile the current landscape of pediatric drug development by (1) outlining the historic regulatory panorama, (2) summarizing the challenges in the development of pediatric drug formulation, and (3) delineating the advantages/disadvantages of using innovative approaches, such as nanomedicines and ATMPs in pediatrics. Moreover, some attention will be given to the role of pharmaceutical technologists and developers in conceiving pediatric medicines.

## 1. Introduction

Pediatrics is the field of medicine that centers on physical, social, and mental health from birth to the end of adolescence [1].

**Figure 1:**
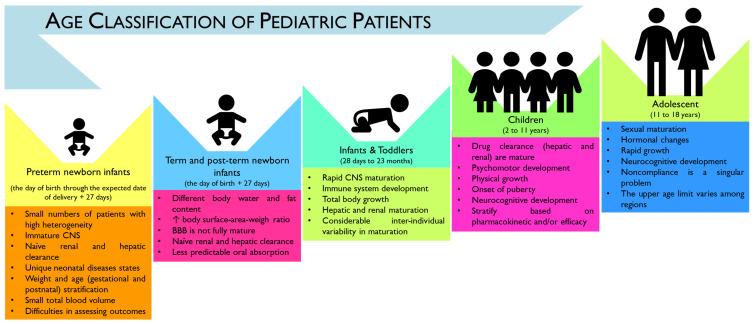
Infographic of the age categorization of pediatric patients according to the International Council for Harmonization (ICH) guideline E11(R1). As summarized, there is considerable heterogeneity in developmental categorization (e.g., physical, cognitive, and psychosocial) across pediatric ages [2]. Central nervous system (CNS), blood–brain barrier (BBB), increase (↑).

The pediatric population can be subcategorized, according to the “International Council for Harmonization” (ICH) topic E11 (CPMP/ICH/2711/99) and the ICH E11(R1), as preterm newborn infants (from the day of birth to the expected date of birth plus 27 days), term and post-term newborn infants (aged from 0 to 27 days), infants and toddlers (with 28 days to 23 months), children (aged between 2 and 11 years old), and adolescents (with age ranges from 12 to 16–18 years old, depending on region) (Figure 1).

However, a considerable overlap can exist across the age subcategories, namely in physical, cognitive, and psychosocial development. Moreover, no consensus seems to exist on the upper age limit of pediatric patients, which may hamper the evaluation and development of age-appropriate treatment plans [3]. In particular, according to the American Academy of Pediatrics (AAP), the upper age limit of pediatrics is considered 21 years, with a proposed subcategorization of adolescence into three main groups: (1) early, represented by adolescents from 11 to 14 years old; (2) middle, for adolescents with ages between 15 and 17 years old; and (3) late adolescence ranging from 18 to 21 years old. However, this age limit has been questioned as increasing evidence has demonstrated that brain development only reaches adult levels of functioning by the third decade of life, which may contribute to the increase in complexity when addressing age-related pathologies and treatments [4].

Historically, the intrinsic heterogeneity in the pediatric population and the reduced number of individuals that can be included per each subcategory in clinical trials may have constituted fatal reasons to dub children as “therapeutic orphans” and for the “off-label” prescription of adult medication to pediatric patients. However, this paradigm has been shifting as it is well recognized that children cannot be considered mini-adults, since the developmental, physiological, and metabolic stages across these two age segments are critically different [5]. The impact on the pharmacokinetics (PK) and pharmacodynamics (PD) of the Active Pharmaceutical Ingredients (API) makes it unreasonable to translate dosage forms and dosage strengths straightforwardly from adults to children [6,7,8].

Therefore, a strategic workforce has been constructed to appropriately reply to disease burden across childhood, addressing the therapeutic deficit and developing age-appropriate formulations, in order to maximize efficacy and design quality, promote safety, minimize risks, and increase patient adherence to treatments [9,10].

Considering the route of administration, the most favored is the oral one. In contrast, the parenteral route remains reserved for more acute conditions, mainly when a quick onset is required [10]. Planning a pediatric oral formulation is challenging, and involves the choice of excipients, dosage form, and palatability [11]. For instance, the choice of dosage form for oral administration depends on the gut function and, thus, on both age and clinical condition [12]. Moreover, the choice of excipients for pediatric drug formulation has been questioned as certain excipients used in adult drug formulation are not adequate for pediatric use, with toxicological risks and safety issues in children [13]. Therefore, the collaboration of the European and the United States Pediatric Formulation Initiatives (PFIs) has resulted in the creation of the “Safety and Toxicity of Excipients for Pediatrics” (STEP) database that aims for the screening of excipients that can appropriately fit pediatric drug formulation [13,14,15]. Furthermore, a set of potentially inappropriate drugs for pediatric use has been released by the “Key Potentially Inappropriate Drugs in Pediatrics” tool, or “KIDs” List, with the primary goal of anticipating risks for adverse drug reactions (ADRs), decreasing severe ADRs, improving the quality of care, decreasing costs, and identifying subjects that need research in the pediatric population [16].

Despite the efforts made in the development of pediatric drug formulation, as well as in age-appropriate medical devices, clinical trials and approved drugs for the pediatric population remain constrained [17,18,19].

Nanotechnology has received enthusiasm among the scientific community, particularly in medicine and pharmaceutical fields, due to its potential to incorporate diagnostic and treatment tools in the same nanocarrier, enhance targetability to specific organs, decrease toxicity, and potentially reduce treatment schedules. At the same time, it provides a tool to increase patient compliance, which is an essential task concerning the pediatric population [20,21,22]. Together with nanomedicine, the advanced therapy medicinal products (ATMPs) have been considered by the European Parliament as the “therapies for the future” [23]. ATMPs are a heterogeneous group of biopharmaceuticals encompassing gene therapy, somatic cell therapy, tissue-engineering, and their combination. These nascent technologies have the potential to reduce or repair disease-causing cells, thereby introducing a curative approach to address the unmet medical needs and highlighting personalized precision medicine [24], with promising applications in the pediatric population [25].

Considering the timely subject and the undoubted shifting in the pediatric drug development paradigm, this literature review aims to outline the historical paradigm in pharmaceutical drug development for the pediatric age, delineating the pros and cons of using innovative therapies, such as nanomedicines and ATMPs, for treating pediatric pathologies, based on a fit-by-design approach, centering its reflection on the role of pharmaceutical technologists and developers in the conception of pediatric medicines.

## 2. Study Conception

A revision of the literature in different databases, such as Pubmed, Web of Sciences, and ScienceDirect, was carried out. Some of the following index terms were included: “advanced therapies medicinal products”, “cell therapy”, “child”, “children”, “gene therapy”, “nanomedicine”, “nanoparticles”, “nanotechnology”, “pediatrics”, “neoplasms”, “tissue engineering”, “drug formulation”, among others. In particular, the following MeSH terms were adopted: Pediatrics; Nanoparticles; Gene therapy; and Cell- and Tissue-Based Therapy. Other core databases were assessed, including https://www.ema.europa.eu/en, https://www.fda.gov/, https://clinicaltrials.gov/, https://www.clinicaltrialsregister.eu/ and https://www.nih.gov/, among others. When appropriate, the boolean operators “AND”, “OR” or “NOT” were applied. The inclusion criteria were the following: articles that contained one of the considered index terms in the title or in the abstract, and were presented preferentially in English. At least one author read the title and the abstract of the manuscripts to select the articles to be included as bibliographic support in this work.

## 3. Pediatric Drug Development: The Paradigm Is Shifting

**Figure 2:**
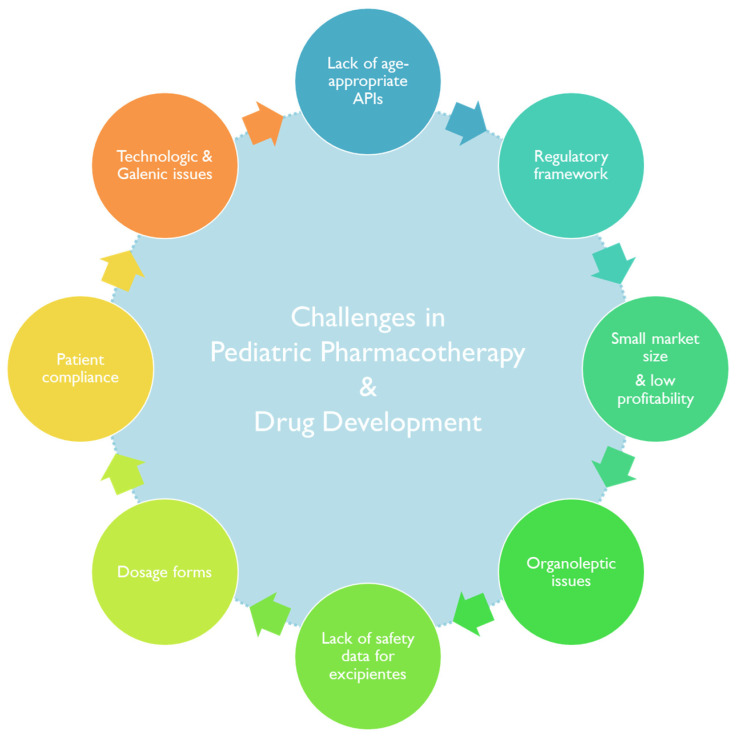
A summary of the factors impacting pharmacotherapy practice and the development of therapeutics aimed at pediatric patients.

The development of pediatric dosage forms and drug formulations has faced particular setbacks (Figure 2) [26,27,28] during the years, with widening repercussions in the off-label prescription of adult medications to pediatric patients [29].

However, the paradigm seems to be shifting, and overdue attention has been invested in overcoming the scarcity of pediatric age-appropriate medicines [30]. In the following section, a historical perspective of the regulatory landscape of pediatric medicines will be given. Next, some notes on the hit-or-miss game in research and financial investment in pediatric drug formulation, followed by an overview of some challenges in pediatric drug pharmacotherapy, will be provided.

### 3.1. Snapshot into the Pediatric Drug Development History

**Figure 3:**
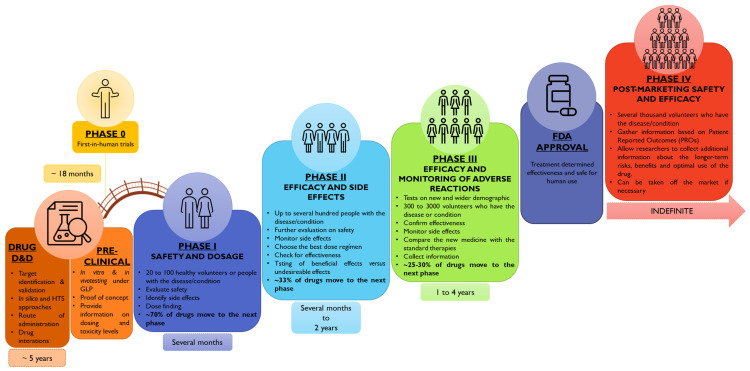
Infographic of the pharmaceutical pipeline and clinical trials timeline. In brief, a screening of potential drug candidates is performed during the drug discovery and development (D&D) step, which takes an average time of five years. The most promising compounds go further to pre-clinical trials under good laboratory practices (GLP), with a possible bridge to the clinical phase by taking advantage of the first-in-human trials (Phase 0), with interesting feedback on dosing and toxicity levels of the most promising candidate, which takes 18 months on average. As part of the investigational new drug (IND) portfolio, the clinical trials can go further, and if the treatment is effective and safe for human use the new drug application (NDA) obtains the approval of the regulatory agency (FDA). After, the pharmacovigilance post-marketing safety and efficacy studies are conducted over time [32,33]. High throughput screening (HTS).

Implementing clinical trials as a new requirement for drug approval has rocked the pharmaceutical pipeline. The “Drug Efficacy Study Implementation Program”, conducted between 1938 and 1962, highlighted the need to reframe the clinical and pharmaceutical pipeline for drug approval. Since then, efforts have been raised to achieve the currently implemented step-by-step-based framework that encompasses drug discovery and development (D&D), pre-clinical studies, bridging first-in-human phase 0 studies, and clinical studies, phase I to IV (Figure 3) [31,32,33,34].

**Figure 4:**
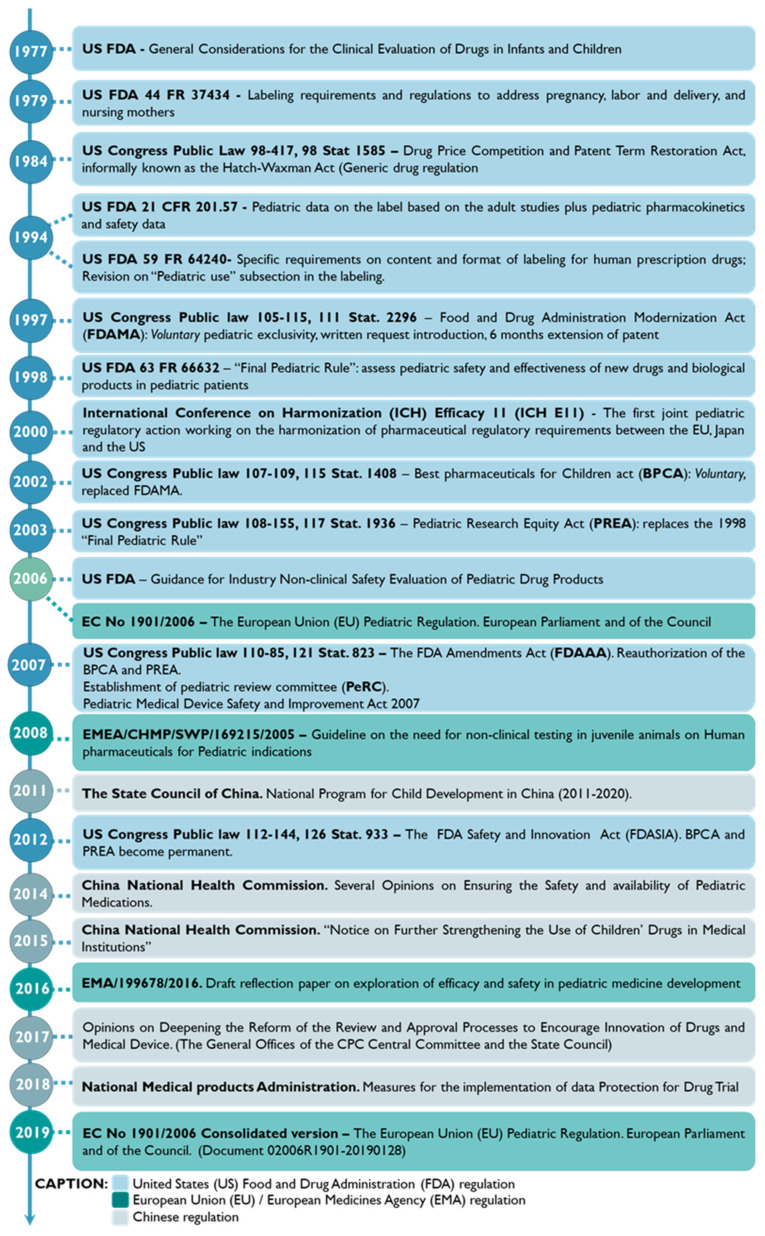
Historical roadmap of the regulation and guidelines developed for clinical studies related to the pediatric population in China, the European Union (EU), and the United States (US) [28,39,40,41,42].

The enrollment of the pediatric population in clinical studies has been steadily increasing [35]. In 1977, the American Academy of Pediatrics (AAP), together with the US Food and Drug Administration (FDA), delivered the “AAP guidelines on clinical studies in pediatric populations” [36]. Later, in 2007, Pediatric Regulation rose in the EU and the US, boosting pediatric drug development through marketing exclusivity incentives [37,38]. Moreover, since 2011, policies encouraging pediatric drug development and distribution have been launched in China [39]. Figure 4 outlines the regulatory background of pediatric drug approval in the US, the EU, and China [28,39,40,41,42].

The Orphan Drug Act seems to be a boon for pediatric medicine [43]. Moreover, supportive initiatives, such as the Pediatric Research Equity Act (PREA), the Best Pharmaceuticals for Children Act (BPCA), and/or the Pediatric Investigation Plan (PIP), have provided a carrot-and-stick approach to pediatric medicine advancements [43,44].

The implementation of EU directive no. 1901/2006 has promoted the accessibility of medicines for individuals under 18 years old, without compromising the access of adults to these products or the well-being of children, requiring the investigation of safety and efficacy and quality on an age-appropriate based approach. Notably, to promote investment in the development of new drug candidates and formulations when preparing a marketing authorization application (MAA), the pharmaceutical industry is requested to include a PIP to address the safety of the medicine for the pediatric population [10].

Despite these achievements, significant heterogeneity in funding sources, pediatric clinical conditions, and study characteristics still impact the participation of the pediatric population in clinical trials [40,45]. Based on a search performed on the https://clinicaltrials.gov/ database, it was possible to detect that among 462,303 registered clinical trials, only 90,920 were designed for children (from birth to 17 years old) (data collected on 14 August 2023). Moreover, in the EU Clinical Trials Register database, EudraCT, from the 43,644 clinical trials reported, only 7229 were conducted in the population less than 18 years old [46]. Moreover, some issues regarding age-appropriate equipment and medical techniques, a “child-friendly” environment, pediatric expert physicians and other health professionals, together with the management of caregivers, may also contribute to limiting the enrollment of the pediatric population in clinical trials [27].

### 3.2. Constrains in Drug Development for Pediatric Patients

#### 3.2.1. Investments in Pediatric Drug Development and Market Trends

Global healthcare spending has been escalating dramatically, which may have been mainly driven by the Coronavirus Disease-19 (COVID-19) pandemic, recent wars, the environmental crisis, inflation, and the increase in food and drug expenses [47,48,49].

In parallel, the pediatric drug market is expected to grow from USD 120.31 billion to USD 179.74 billion from 2023 to 2028 at a Compound Annual Growth Rate (CAGR) of 8.36% during the forecast period [50]. This expected growth may result from multiple factors, particularly the increasing rise in the birth rate compared to previous years and the number of fatal pediatric diseases that continue to contribute to deaths in pediatric age. Infectious diseases such as pneumonia, diarrhea, malaria, preterm birth, or intrapartum complications continue to represent the principal causes of death among children under 5 years of age worldwide. According to the United Nations International Children’s Emergency Fund (UNICEF) 2020 Report, 5.0 million children under five died in 2020, demanding the need for efficient treatments and socioeconomic incentives.

**Figure 5:**
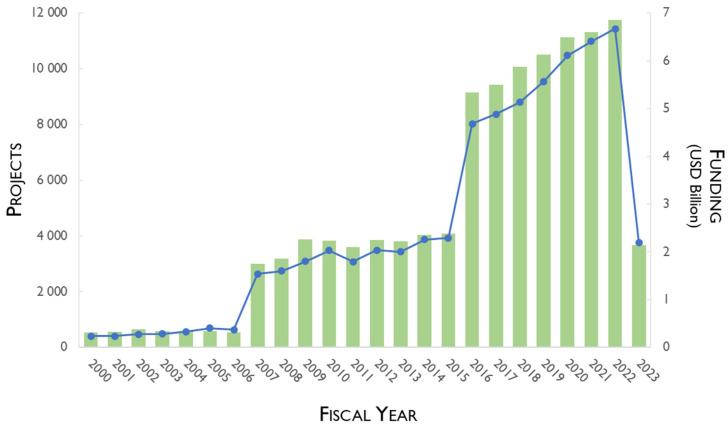
Overview of the granted projects (green bars) and the financial support (blue line) sponsored by the National Institutes of Health (NIH) that address the pediatric population, between 2000 and 2 August 2023 [52].

The pediatric research portfolio has been indicated as vulnerable with a high grade of uncertain fate [51]. However, interestingly, the National Institutes of Health (NIH) has given strategic attention to pediatric research since 2000, which is evidenced by the increase in the number of supported projects and financial investment, with some fluctuations in 2011 (Figure 5) [52,53]. Moreover, considering the quickly changing healthcare needs and pediatric diseases, the priorities for federal pediatric research support may need some adjustments [53].

Among the different financed projects, the networks related to pediatric Human Immunodeficiency Virus (HIV)/Acquired Immunodeficiency Syndrome (AIDS) infections and childhood cancer are examples of clearly established teams studying and developing therapies for children with life-threatening diseases [54]. Another example is the Conect4children, a European network intended to facilitate the development of new medicines for pediatric populations. This collaborative group has recently published recommendations to improve pediatric clinical research based on the outcomes of COVID-19 [55].

Another critical factor is that academic institutions face increasing constraints, particularly impacting the education of junior physician-scientists, the uneven distribution of pediatricians among the different subjects, the increasing costs in research and development, and limited reimbursement due to the reduced percentage of the pediatric population. Consequently, to obviate these challenges, a pediatric physician workforce together with clinical pharmacologists has been encouraged [49,56].

#### 3.2.2. Lack of Approved Active Principal Ingredients for Pediatric-Age Patients

Pediatricians continue to demand more safe medications [16,29,57], especially in the field of molecular target antineoplastic drugs. A recent report revealed that of 103 drugs approved for adult patients, only 19 were approved for pediatric patients [18]. Moreover, it was reported that pediatric labeling was not established for 78 medications out of 189 products under pediatric exclusivity (1998–2012), corresponding to a failure rate of 42% [58]. Additionally, a lag phase between adult and pediatric drug approval remains and can take over a decade to resolve [57,59].

Therefore, off-label prescription seems to persist as a rule and not an exception in pediatrics [60]. Recently, Allen et al. [61] reported that 38.1% of the medications prescribed to pediatric patients remain off-label. It is remarkably evident in younger populations, especially neonates, with an off-label prescription rating of at least 26% [61]. Moreover, the prevalence of pediatric off-label drug prescriptions has been estimated to range from 2.7 to 51.2% in outpatients and 9.0 to 79.0% in inpatients, respectively [60]. However, the off-label use of drugs could be unsuitable, deprived of therapeutic benefits for pediatric patients, or responsible for adverse events. The most recent data/evidence presented in the Clinical Practice Guidelines (CPGs) could contribute to mitigate the risk of irrational pharmaceutical use and the liability associated with the off-label use of drugs [59].

Moreover, since 2017, the Institute for Advanced Clinical Trials (I-ACT) for Children has brought together leading specialists and biopharmaceutical companies to promote the timely availability of innovative drugs for children [29]. Additionally, in 2019, the Pediatric Innovation Research Forum advocated the routine enrollment of adolescents in phase III clinical trials [62].

#### 3.2.3. Lack of Pharmacokinetic and Pharmacodynamic Data

Age-related effects on drug PK and PD profiles are not fully understood [63]. PD is generally defined as the effect of a drug on the body and is often characterized as a drug response. On the other hand, PK is classified as the effect of the body on a drug [64]. Rapid growth and development during childhood exacerbate dosing issues, with dosages of specific formulations fluctuating 100-fold [65].

The relationship between drug exposure and PD endpoints seems to be weakly studied in children [64]. The FDA has proposed a guideline (September 2022) entitled “General Clinical Pharmacology Considerations for Pediatric Studies of Drugs, Including Biological Products”, aiming to address clinical pharmacology considerations of any planned pediatric study, whether or not it is conducted under BPCA or PREA [66]. Based on this, clinical pharmacology studies should be conducted in a specific pediatric population that presents a particular pathology for which the drug is intended or, in rare instances, in those at risk of this disease [67].

PK parameters are particularly articulated with the measurement of area under the curve (AUC), maximum concentration (C_max_), clearance, half-life, and volume of distribution (V_d_), which in turn reflect the absorption, distribution, metabolism, and excretion (ADME). These parameters tend to differ across the different age groups, with relevant emphasis in the pediatric population [68]. Therefore, understanding ADME differences may contribute to ensuring effective and safe therapies in pediatric populations [69]. Moreover, PK measures may consider growth parameters such as age, weight, or body surface area (BSA). The heterogeneity across the different subpopulations in pediatric ages is particularly challenging, with remarkable variability in PK inside the same subgroup. For instance, the weight range across pediatric patients (from 400/500 g to 70 Kg) can limit adequate stratification according to age or developmental stage in clinical trials’ design and analysis plans [64,70].

**Figure 6:**
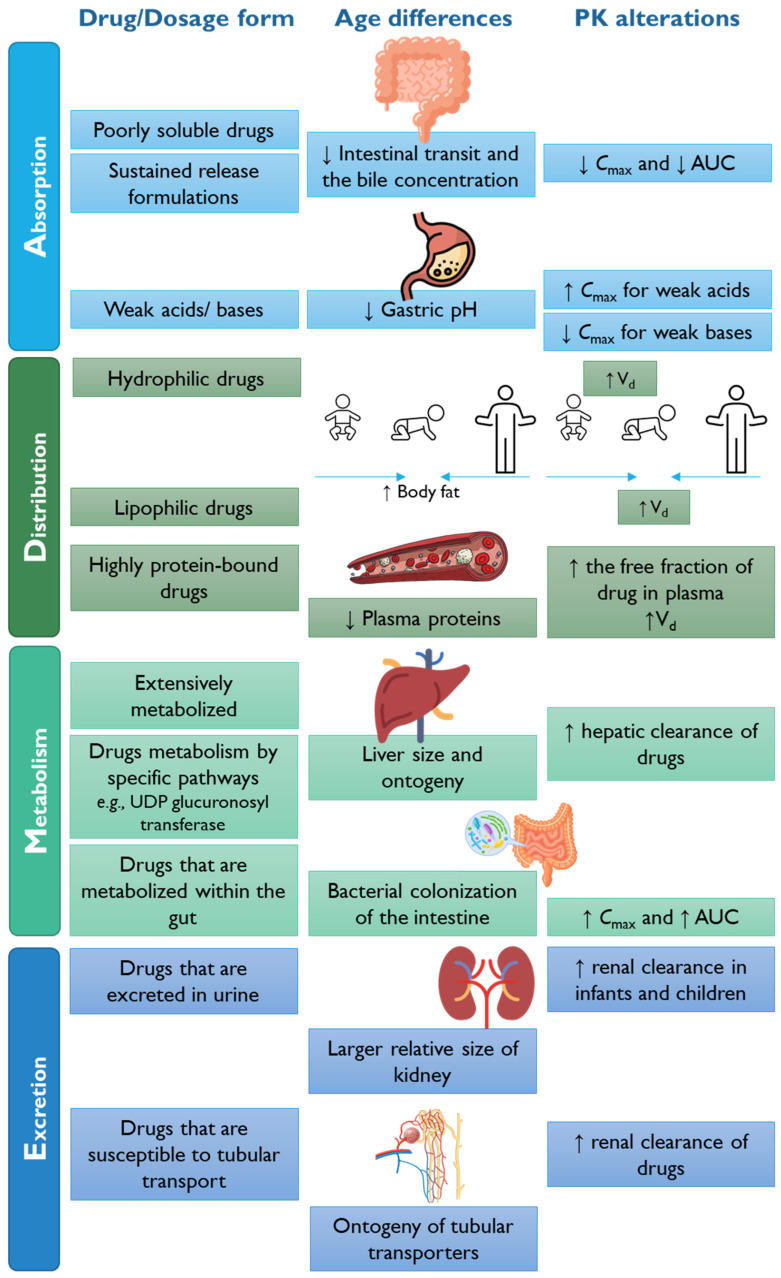
Summary of pharmacokinetic differences in the pediatric population compared with adults [69]. Area under the curve (AUC); maximum concentration (C_max_); volume of distribution (V_d_); increase (↑); decrease (↓).

Moreover, genetic polymorphisms have been associated with drug disposition and response variability, specifically in drug metabolic enzymes. Understanding genetic polymorphisms may be a key factor in providing personalized dosing in pediatrics [70]. Figure 6 summarizes the elements, e.g., drug physicochemical properties, dosage forms, and age-dependent anatomical/physiological characteristics, that can impact the ADME and, consequently, the PK profile of APIs in the pediatric population, as reviewed previously elsewhere [69].

In brief, changes in the pediatric population that can affect oral absorption (A) include gastric acidity, rates of gastric and intestinal emptying, the surface area of the absorption site, gastrointestinal drug-metabolizing enzyme systems and permeability, biliary function, and transporter expression. Drug absorption in childhood is highly affected by changes in gastric pH, which is neutral at birth, decreases two to three days after birth, and continues for weeks or years until adulthood. Moreover, the gastric emptying time is slower in six- to eight-month-old infants, as the neuroregulation of gastric motility is immature [71]. Furthermore, the absorption of drugs administered intramuscularly, subcutaneously, or percutaneously can also be impacted by different water content and degrees of vascularization across the different pediatric subpopulations [63].

Drug distribution (D) can be affected by changes in body composition, particularly in total body water and adipose tissue content, as well as by changes in plasma protein and tissue binding. Another important parameter is the difference between the blood flow to specific organs, like the brain, in the pediatric and adult populations [66].

The metabolism (M), biovailability, and elimination of a drug can also be impacted in different pediatric subgroups and can depend on the degree to which intestinal and hepatic metabolic processes are implicated [72]. For example, the most associated drug metabolic enzyme, CYP3A, appears to be abundantly expressed in the small intestine of adults. However, the levels of this enzyme across different pediatric age subgroups remain unclear [69]. The ontogeny of drug metabolism in newborns, infants, and children has been recently included in modeling approaches to predict drug elimination in these groups [73,74]. Moreover, the microbiota can also impact drug metabolism in pediatric subgroups compared to adults [75,76,77].

The excretion/elimination (E) of unchanged parent drugs can occur predominantly via the kidneys, through glomerular filtration, tubular secretion, and reabsorption, and can be affected across different pediatric ages. For instance, generally, uncharged drugs have lower excretion levels by the kidneys in newborns due to the immaturity of renal function. However, some drugs, such as levetiracetam, cimetidine, and cetirizine, have demonstrated a similar or greater renal excretion rate in infants and preschool children than in adults [69]. Moreover, the urinary pH can influence the reabsorption of weak acids or bases. Therefore, as the urinary pH is lower in infants compared to adults, the reabsorption of weakly acidic drugs may increase [78]. Furthermore, some drugs can suffer hepatic biotransformation to both inactive and active metabolites [79]. However, information on the developmental changes in the biliary excretion of drugs remains scarce [80]. Interestingly, in a study developed by Johnson et al. [80], they revealed through in silico PKPD modeling that the ontogeny of biliary excretion for drugs used in pediatrics, such as azithromycin, ceftriaxone, digoxin, and buprenorphine, attains adult levels at birth or within a few months of postnatal age [80].

Other factors impacting age-related issues in drug PK and PD in pediatrics are the immature secretion of bile and pancreatic fluids, as reviewed previously [34]. In brief, in neonates, inadequate levels of bile salt in the ileum may determine the reduced absorption of fat-soluble vitamins, like vitamin D or E, leading to the need for dose adjustments when administering fat-soluble substances for this age group. After a few months, the postnatal maturation of bile salt may grant that the infants can efficiently absorb fat-soluble compounds [34].

Therefore, to circumvent some of the challenges described above and leverage pediatric pharmacotherapy, conducting all PK studies in the target pediatric population would be of interest. However, in order to protect children from unnecessary in vivo studies, a growing demand for alternative tools that can accurately mimic the typical PK-related processes in pediatric patients has grown. To address this topic, the development of in vitro–in vivo correlation (IVIVC) approaches [81], or in silico tools such as physiologically based pharmacokinetic–pharmacodynamic (PBPK-PD) modeling approaches, have been widely explored [67,74,82,83,84,85,86,87,88,89]. Accordingly, PBPK modeling has been readily applied for dose regimen selection in various pediatric patient groups in “learn and confirm”-based studies [90].

Nevertheless, more validated system data are needed. A careful evaluation of the parameters mentioned above, particularly PK and PD, should be considered to fit an adequate administration route and dosage form in pediatrics [71].

#### 3.2.4. Administration Route and Pharmaceutical Dosage Forms in Pediatrics

Preparing and selecting the most appropriate dosage form that ensures safe administration and adherence to medications for pediatric age is particularly challenging and there is no ‘one-size-fits-all’ approach [91]. Available pharmaceutical dosage forms can be divided into types of dosage form and the intended route of administration [92].

There are various routes of drug administration for pediatric patients, such as oral [93,94], dermal–transdermal [95,96], rectal [97], intramuscular [98], parenteral [99], intrapulmonary [100], and inhalation [101,102]. Generally, the ideal dosage form of high-quality pediatric medicines should take into consideration (1) that the amount of the API is adjusted to the age needs of the child, and thus the intended dose volume and size should be appropriate for the target age group; (2) the acceptability of the dosage form; (3) the palatability of the API, which may influence the choice of dosage form and its design, it being preferable that the dosage form is palatable in itself without any need for further modification, although in some cases the adding of excipients in the formulation is required for taste-masking purposes; (4) minimum dosing frequency, to guarantee the adherence to the dosing scheme both by caregivers and by older children; (5) the end-user needs, for instance, water accessibility, which is important when a medicine needs to be dissolved, diluted, or dispersed prior to administration; or, for example, (6) the regional and cultural differences that may impact the preferred tastes and flavors [12].

In pediatrics, the oral route of administration tends to be the most commonly used. Therefore, different oral dosage forms intended for pediatric oral administration have been employed and studied, namely solid dosage forms, such as tablets, capsules, orodispersible formulations, powders for reconstitution, and chewable tablets, as well as liquid dosage forms, like solutions, suspensions, elixirs, and syrups [103]. When developing an oral dosage form it is pivotal to consider age-related gut function and health stage [12]. Despite the advantages of solid dosage forms, particularly their long-term stability, manufacturing flexibility, low production costs [94,103], acceptability in infants, and suitability for school-age children and adolescents [92], they appear to be seldom used in pediatric practice [104]. Instead, liquid dosage forms are the most commonly used in ages lower than 5 years old due to the facility for swallowing and dose adjustment [11]. In spite of liquid dosage forms being preferable, many of them are not labeled for pediatric populations, and those labeled are not available in the appropriate dosage forms. To overcome these issues, some dosage forms, such as tablets or capsules, are used to prepare “especially” or “extemporaneously” liquid or powder dosage forms [92]. However, this may lead to dosing errors due to poor division or an extemporaneous way of dispensing, which is even more critical for antibiotics widely prescribed to the pediatric population [92].

Additionally, the dosing volume is also of significant importance when determining acceptability. Target volumes are ≤5 mL and ≤10 mL for children under 5 years and children above the age of 5 years, respectively [11,103]. However, according to the EMA draft guidance, maximum volumes of 5 mL or 10 mL were recommended for children under 4 years or between 4 and 12 years, respectively (EMA/CHMP/QWP/180157/2011). Regarding stability, many liquid preparations require refrigeration at temperatures of 5 °C (±3 °C), which may be a difficulty in developing countries with limited access to refrigeration. Another concern with liquid formulations is their relatively shorter shelf life [103].

In cases of vomiting, nausea, or palatability issues, the oral route can be replaced by the rectal route [60]. Rectal administration can be used when aiming for local (e.g., laxative and anti-inflammatory) and systemic (e.g., antipyretic and anticonvulsive) effects in all age groups. However, limited absorption and bioavailability for many APIs, unpredictably delayed absorption, and uncomfortable administration may hinder its choice [12].

The parenteral route is used in pediatric ages, particularly in acute situations, to ensure a rapid onset of action and high bioavailability in treatment. Some parenteral preparations can be administered by the subcutaneous and intramuscular routes. However, this type of administration requires trained professionals and is a more invasive process, with risks of blood-borne infections, injury, and pain induced by injections. To overcome some of these issues, the use of the buccal route can be much more suitable. For example, pediatric patients with cancer suffer from severe breakthrough episodes of pain and need prompt and efficient pain treatment. In these cases, the oral route does not fulfill the need for an immediate response to eliminate discomfort. Buccal medication delivery is an appealing administration route for pediatric pain management, with a quick onset of action and no hepatic first-pass metabolism. However, physiological considerations, regulatory expectations, and formulation development considerations have limited its broad translation [105,106].

Pharmaceutical dosage forms intended for dermal (or cutaneous) administration are tailored to promote a local effect. They can encompass liquid preparations, such as lotions or shampoos; semi-solid preparations, like ointments or creams; as well as solid preparations, e.g., powders [12]. Accidental systemic absorption through the dermis of APIs could have a dramatic impact on preterm neonates, as the stratum corneum is deficient, and in children as the volume of distribution per unit of skin area is lower [12]. Importantly, the use of ethanol should be avoided as an excipient in preparations intended to be used in very young children because ethanol may dehydrate the skin and cause pain [12]. On the other hand, transdermal administration, for example using patches, is intended for systemic delivery of APIs that are capable of diffusion through the stratum corneum [12]. Their relevance in children has been recently reviewed by Delgado-Charro & Guy [95]. Depending on the dosage form, the assessment of excipient safety is of the utmost importance (see Section 3.2.5). Although not appropriate for all drugs, various transdermal patches containing active drug ingredients such as fentanyl, clonidine, or scopolamine have been exploited for pediatric applications [95]. According to the EMA report, EMEA/CHMP/PEG/194810/2005, the use of transdermal patches (“needle-free infusion”) allows continuous and painless active drug permeation over hours or even days, contributing to the increase of patient compliance.

The pulmonary administration of medicines by inhalation has traditionally been used to obtain a local effect. Additionally, it also presents the potential for systemic delivery. Preparations for inhalation include liquids for nebulization, pressurized metered dose inhalers (MDIs), and dry powder inhalers (DPIs) [107]. Inhalation products are mostly used to treat asthma and Chronic Obstructive Pulmonary Disease (COPD) in pediatric patients.

Although progress has been achieved in drug formulation for the pediatric population, some problems remain to be solved. The design of pediatric drug formulation needs to be based on the patient-centric drug product design process (PCDPD), namely patient, drug, and drug product characteristics, that are translated into a Quality Target Product Profile (QTPP) to drive the pharmaceutical product design process [108,109]. Moreover, assessing the safety of excipients used in the formulation of pediatric pharmacotherapy is crucial.

#### 3.2.5. Excipients

Excipients are important constituents of medicines that are anticipated to have no direct biological or therapeutic effect [110]. Excipients can be classified as diluents or fillers, binders, disintegrants, lubricants, glidants, preservatives, antioxidants, sweeteners, surfactants, taste maskers, coloring agents, flavoring agents, and coating agents, aiming to improve product performance, e.g., stability and bioavailability, to ensure that the desired properties of the formulation and patient compliance are accomplished [111].

According to Reker et al., based on the Pillbox database (https://pillbox.nlm.nih.gov), an open-access on-line resource that compiles data from the FDA, the NIH, pharmaceutical companies, and the Department of Veterans Affairs, an average tablet or capsule contains 8.8 inactive ingredients [110]. The possibility of having more than 23 alternative combinations of inactive ingredients to deliver the same APIs highlights the diversity of available alternatives to medications in terms of their inactive ingredient portion, and the crucial need to study the differences between those alternatives further. In fact, despite excipients are commonly classified as safe, some adverse reaction-associated inactive ingredients (ARAIIs) events in the form of allergies or intolerances have been reported [110]. Moreover, the use of excipients across different ages may present different tolerability and safety profiles, particularly in the pediatric age, due to the ontogeny of pediatric organs, which influences dose-dependent adverse events [14,112,113]. Of relevance are propylene glycol (PG) and ethanol, which cannot be metabolized in the same manner as in adults due to the immaturity of the organs, particularly in young children [114].

The lack of data regarding the use of excipients in pediatric drug formulation has encouraged the “European Pediatric Formulation Initiative” (EuPFI) to develop the STEP open-access database. The main goal of this initiative is to compile data from peer-reviewed journals, government reports, and other databases, enabling user-friendly and rapid access to pharmacologic, toxicologic, and safety information of excipients [115].

The evaluation of certain excipients have been prioritized, such as benzalkonium chloride, benzoic acid and benzoates, benzylalcohol, cyclodextrins, ethanol, and PG, as some reports had revealed that they cause more damage and side effects in the pediatric population [14,116]. On the other hand, Polyethylene glycol (PEG) has been extensively utilized in pharmaceutical formulations, not only as an API (e.g., PEG 3350 or PEG 4000 in colonoscopy preparations or laxative solutions), but more commonly as a multifunctional excipient due to its broad range of physicochemical properties and applications [117]. In addition, many PEG-modified (PEGylated) drugs are being developed and approved for marketing, with a broad spectrum of applications, e.g., cancer, hepatitis, or immunotherapy. For example, Oncaspar^®^, a pegylated form of the enzyme asparaginase (PEG-ASNase), was approved by the FDA in 1994 for pediatric Acute Lymphoblastic Leukemia (ALL) [118]. Allergic reactions to PEG are not commonly described, but when reported they could be severe or even fatal [119]. For example, PEG allergic reactions were reported in 13% of children aged from 1 to 17 years old treated with PEG-ASNase based-formulations [120]. These safety issues become more prominent due to the presence of PEG in the messenger ribonucleotide acid (mRNA) COVID-19 vaccine [121,122].

#### 3.2.6. Pediatric Patient Acceptability

Allergic medication non-adherence has been considered by the World Health Organization (WHO) as a global public problem with significant consequences [123]. It is a multifaceted problem involving the patients, caregivers, and interdisciplinary healthcare team [124]. In the 2013 “Guideline on pharmaceutical development of medicines for pediatric use”, the EMA emphasized the need to consider the acceptability of pediatric medicines as part of clinical studies (EMA/CHMP/QWP/805880/2012 Rev. 2) with the evaluation of clinical outcome assessments (COAs) being of greatest importance to capture patient and caregiver perspectives on pediatric medicine development [125].

Non-adherence to medication is particularly challenging to circumvent in the pediatric population, as it depends on multiple factors such as age, cultural background, socioeconomic status, health literacy, and family structure [123,124].

Some issues directly related to formulation have been indicated as contributing to pediatric patient non-adherence to treatments, namely the recalcitrance and organoleptic properties of the formulation (palatability), particularly the unpleasant taste of some APIs [116,126]. To overcome these issues, some factors should be taken into consideration and included into the QTPPs, namely an absence of unpleasant tastes and smells, acceptable mouthfeel (viscosity, grittiness), and appearance (visual aspect, size and shape, packaging) [10,127].

In addition, the pediatric patient and caregivers’ acceptability and adherence rates may also be influenced by the type of dosing device (spoon, oral syringe) or therapeutic schemes [128]. Acceptability is even more critical when referring to young children dependent on a caregiver for drug administration [126,129], who sometimes tend to manipulate the dosage form without clinical indication, which may lead to altered bioavailability and adverse drug reactions, reinforcing the need to perform acceptability tests during (early) pediatric clinical trials and pediatric formulation development with an internationally harmonized scheme [116,126].

The application of nanotechnology can potentially offer interesting tools that could help to overcome some of the previously described issues [21,130]. Hence, the following section aims to provide an overview of the potential for applying nanotechnology, particularly nanoparticles, in pediatric medicine.

## 4. Nanomedicine for Pediatric Healthcare

**Figure 7:**
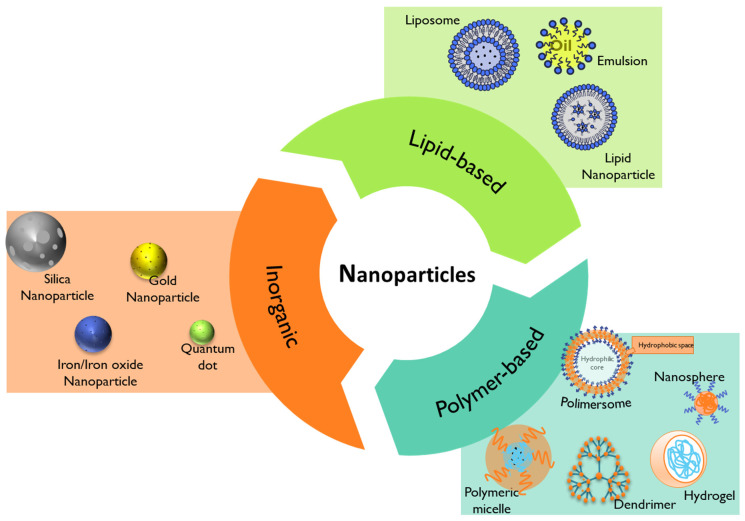
Summary of the different types of nanoparticles that can be used in nanomedicine.

Nanomedicine has emerged through the conjugation of two main fields, namely nanotechnology and medicine. The European Technology Platform on Nanomedicine (ETPN) defined the term nanomedicine as the use of nanotechnology to achieve advances in healthcare by exploiting unique bio and physicochemical properties of materials at the nano scale [131]. On the other hand, the EMA refers to nanomedicine as the application of nanosized components with specific advantageous properties, such as better targeting and bioavailability of therapeutics, new modes of therapeutic action, and nanostructured surfaces/scaffolds for engineered tissues [132]. Among the most studied nanoparticles intended for the prophylaxis, diagnosis, and treatment of diseases are inorganic, lipid-based, and polymeric-based nanoparticles (Figure 7) [20].

In the field of pediatric medicine, the use of nanomedicine has offered innovative solutions for the diagnosis and treatment of various conditions, particularly in cancer [133,134,135], infection [136], dentistry [137], dermatology [138], and nutrition [139].

### 4.1. Lipid-Based Nanoparticles

Lipid-based nanoparticles comprise liposomes, lipid nanoparticles, and emulsions (Figure 7) [118]. Their advantageous properties, like biocompatibility, formulation simplicity, and payload flexibility, make them the most highly approved nanomedicines by the FDA [20,118].

Liposomes are typically composed of phospholipids, which can form unilamellar and multilamellar vesicular structures which allow the delivery of hydrophilic, hydrophobic, and lipophilic drugs in the same system. Liposomes can be modified to extend their circulation and enhance delivery, avoiding rapid detection from the reticuloendothelial system (RES) [118].

Nano-emulsions are heterogeneous oil-in-water or water-in-oil emulsions mainly formed by oil droplets containing the API, stabilized by surfactants and cosurfactants and dispersed in an aqueous external phase [20]. They are usually prepared using Generally Recognized as Safe (GRAS)-grade excipients approved by the FDA [140], and possess high loading capacity for lipophilic APIs with some thermodynamically reported instabilities [141].

The development of next-generation lipid nanoparticles, namely solid lipid nanoparticles (SLNs) and nanostructured lipid carriers (NLCs), has emerged to overcome some limitations of the conventional lipid-based nanosystems [20,142]. Lipid-based nanoparticles like SLN and NLCs can offer the targeted delivery of drugs, increase the bioavailability of hydrophobic drugs, and protect sensitive active compounds [20].

Lipid-based nanoparticles have been widely investigated for various applications, namely in cancer [143,144] and more recently in the formulations of the mRNA COVID-19 nano-vaccines [145], with some of them approved by the FDA for different therapeutic purposes (Table 1).

Among these, liposomes are the most widely studied in pediatrics, and transversal variations in the PK parameters have been registered between the adult and the pediatric populations (Table 2) [161,162].

Furthermore, significant differences between the participation of children (birth–17 years) versus adults in clinical trials using liposomes (clinicalTrial.gov database, data collected by 7 August 2023) have been registered. In fact, of 285 clinical trials that are currently recruiting or not yet recruiting, only 31 include liposomes in pediatrics (birth–17 years), with the majority of them addressing cancer treatment (Table 3).

Other types of lipid nanoparticles, such as in situ self-assembly nanoparticles (ISNPs), have been investigated. For example, child-friendly Lopinavir/Ritonavir pediatric granules utilizing ISNPs were developed. In vivo pre-clinical data demonstrated that the orally administered formulation improved lopinavir bioavailability and concentration in the brain and lymphoid tissues, the target sites of the HIV [163]. In another study, Rodríguez-Nogales et al. formulated nano-assemblies using squalenoyl-gemcitabine and alkyl-lysophospholipid edelfosine with a nanoprecipitation method. Their results revealed that the 50 nm nanoparticles presented a high uptake by human osteosarcoma cells, resulting in antitumoral activity and enhanced gemcitabine and edelfosine pharmacokinetic profiles [164].

### 4.2. Polymer-Based Nanoparticles

**Figure 8:**
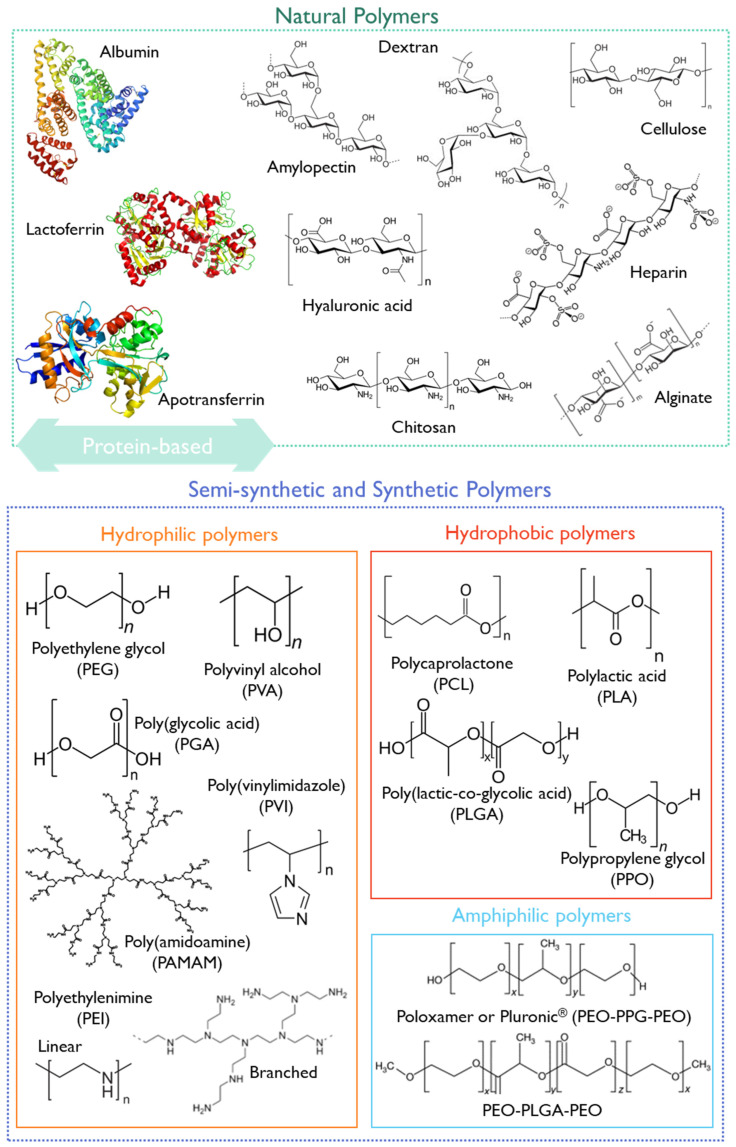
Structural representation of some natural, semi-synthetic, and synthetic polymers.

Polymer-based nanoparticles are colloidal systems made up of natural, semi-synthetic, or synthetic polymers (Figure 8), allowing for a wide variety of possible architectures and characteristics [20,162]. They include dendrimers, polymeric micelles, polymersomes, nanospheres, and nanogels (Figure 7) with diverse clinical applications [20]. Usually, natural polymers present fewer toxic effects than synthetic polymers [165].

They can be biodegradable or non-biodegradable. As biodegradable polymers undergo biodegradation in vivo through enzymatic or non-enzymatic pathways producing biocompatible or harmless by-products, they have been preferred in nanomedicine, particularly for pediatrics [162]. The performance of polymeric biodegradable formulations can be improved by (1) using FDA-approved biodegradable polymers, (2) administering the formulations in situ, (3) using combined therapies, such as immunotherapy or radiotherapy, and (4) applying the on-demand delivery of molecularly targeted agents [166].

Some examples of biodegradable polymers are polysaccharides, such as hyaluronic acid, chitosan, dextrin, or alginate (Figure 8).

Chitosan is a natural biocompatible and biodegradable cationic polymer with low toxicity. It is based on deacetylated chitin [167] obtained from crustaceans, insects, squibs-centric diatoms, or fungi [168]. At an acidic pH, chitosan presents a high density of positive charges that deliver mucoadhesive properties, and a suitable environment for complexing anionic polymers or nucleic acids [169]. Moreover, it can entrap poorly water-soluble drugs, combining antimicrobial, anti-inflammatory, and wound-healing effects [170]. This polymer has been classified by the FDA as GRAS [171], and is approved as a biomaterial for use in tissue engineering and drug delivery applications [172]. Furthermore, chitosan has been applied in developing pediatric formulations (Table 4), and some chitosan formulations underwent clinical trials, as summarized in Table 5.

However, concerns regarding the source, purity, and immunogenicity of chitosan have hampered its approval for pharmaceutical applications [172].

Hyaluronic acid (HA) is a mucopolysaccharide present in the extracellular matrix, synovial fluid, and connective tissues, consisting of D-glucuronic acid and (1-b-3) N-acetyl-D-glucosamine alternating units (Figure 8) [166]. HA is biocompatible, non-immunogenic, and biodegradable, and presents a viscoelastic nature, making it suitable for nanomedicine applications [166]. Cluster of differentiation-44 (CD44) is a main receptor of HA and is overexpressed in solid tumors, making it suitable for cancer-targeting purposes [178]. Due to its versatile properties, HA has been studied for pediatric drug formulations, aiming at increased patient compliance through the modification of the dosage form or by decreasing the dosing frequency [179,180,181]. Moreover, HA has already undergone clinical trials, with 91 registered entries addressing the pediatric population (birth to 17 years).

Another group of natural polymers is the protein-based biomaterials, such as albumin, lactoferrin, or apotransferrin (Figure 8).

Albumin is a water-soluble globular protein present in ca. 50% of the total plasma body mass. Due to its hemocompatibility, albumin has been applied for intravenous gene and drug delivery. Consequently, an albumin-based nanosystem for the delivery of paclitaxel (Abraxane^®^) received FDA approval in 2005. According to the information approved by the FDA in 2020 (Reference ID: 4661467), the safety and effectiveness of Abraxane^®^ have not been established in pediatric patients so far. However, in 2013, a Phase 1/2 clinical trial (NCT01962103) was begun aiming to find the safe dose of nab-paclitaxel, Abraxane^®^, in children with solid tumors, and to see if it could constitute a treatment for children and young adults with solid tumors (1 ≤ 18 years old in Phase 1 and 2 ≤ 24 years old in Phase 2).

Lactoferrin (LF) is a natural cationic iron-binding glycoprotein present in milk, with antiviral, anti-inflammatory, antioxidant, anti-cancer, and immune-stimulating effects [182,183]. LF receptors are known to be overexpressed in cancer and endothelial brain cells, making them suitable for active tumor targeting or crossing the blood–brain barrier (BBB) via receptor-mediated transcytosis for brain delivery. In addition, LF-based nanocarriers were found to have a pH-dependent release profile. At an acidic pH, a faster drug release is observed, which could increase drug release in acidic sites such as the tumor tissue microenvironment and could enhance the therapeutic efficacy of the encapsulated hydrophobic active molecules [182,184]. Commercial preparations of bovine lactoferrin, recognized as GRAS by the FDA, are commonly used in in vitro and in vivo testing. Recently, recombinant human lactoferrin has also become available [185]. Ahmed et al. [186] developed LF-based nanoparticles containing carboplatin to address retinoblastoma in children. Apotransferrin-based nanoparticles were also prepared as they are also implicated in iron transport [186,187]. In another study, Narayana et al. developed carboplatin and etoposide-loaded LF nanoparticles to address retinoblastoma treatment in vitro [188].

Semi-synthetic or synthetic polymers have also been exploited for pediatric applications. The FDA-approved synthetic polymer PEG is widely used due to its biocompatibility and biodegradability [162,189]. It is often combined with other more hydrophobic polymers or other API nanocarriers since it provides stealth properties and improves the pharmacological properties of nanomedicines. However, some allergic reactions were reported when using PEG as an excipient in pediatric drug formulation, which may limit its use (as reported above, Section 3.2.5).

Polycaprolactone (PCL) is recognized as non-toxic and suitable for controlled/sustained drug and vaccine delivery owing to its high permeability in relation to drugs [166]. Conjugates of PLC with PEG have recently been reviewed [190]. Krishnan et al. produced PEG-PCL nanoparticles using the nanoprecipitation method, aiming at treating leukemia in the pediatric population. The in vivo results have demonstrated improved life quality and survival in mice in the dexamethasone-loaded nanoparticles group compared to the free drug group [191].

The FDA-approved polymer poly lactic-co-glycolic acid (PLGA) has shown suitable properties for drug delivery, with improved circulation time and permeability. PLGA is an aliphatic polyester polymer that comprises a synthetic copolymer of lactic acid (α-hydroxy propanoic acid) and glycolic acid (hydroxy acetic acid) with demonstrated potential for drug delivery and tissue engineering scaffolds [192]. The 50:50 ratio of lactic to glycolic acid monomers and molecular weight PLGA (3–9 kDa) have been associated with decreased half-time and fastest degradation [161]. PLGA-PEG nanoparticles have been synthesized and decorated with a CD133 aptamer to target salinomycin delivery to CD133^+^ pediatric osteosarcoma cancer stem cells [193].

Other synthetic polymers (Figure 8), such as polyethyleneimine (PEI), poly(vinylimidazole) (PVI), or poly(amidoamine) (PAMAM), will be discussed in more detail in Section 5.3 due to their unique properties for gene delivery.

Due to their versatility, the arrangement of different polymers can result in different nanoparticle architectures. The following sections will give a brief overview of the use of polymeric micelles and dendrimers in pediatric nanomedicine.

#### 4.2.1. Polymeric Micelles

Polymeric micelles (Figure 7) exhibit versatile features as drug carriers and as active ingredients [194,195]. Polymeric micelles are usually characterized as a core–shell structures developed through the self-assembly of amphiphilic block copolymers in an aqueous solution, with attractive flexibility for functionalization [196]. For instance, the use of amphiphilic-block co-polymers, such as Pluronic^®^ (Figure 8) and Tetronic^®^ surfactants, can form polymeric micelles above the critical micellar concentration/temperature with singular features [196,197]. The use of Pluronic^®^ mixed micelles based on F127 and P123 surfactants was reported for curcumin incorporation to treat pediatric osteosarcoma [198].

To date, some polymeric micelles-based nanomedicines have reached the market, such as Genexol-PM^®^, Nanoxel-PM^TM^, and Paclical^®^ [199,200]. Genexol-PM^®^ is a polymeric micellar formulation of paclitaxel, composed of the low-molecular-weight amphiphilic diblock copolymer, monomethoxy poly (ethylene glycol)-block-poly(D,L-lactide) (mPEG-PDLLA) [201], that was approved for the treatment of metastatic breast cancer, non-small cell lung cancer (NSCLC), and ovarian cancer in South Korea, Philippines, India, and Vietnam [199]. On the other hand, Nanoxel^TM^, DO/NDR/02, is a micellar formulation that consists of a di-block copolymer (poly-(vinylpyrrolidone)-b–poly-(N-isopropyl acrylamide) (PVP-b-PNIPAAM) with paclitaxel as the API [200]. It is a liquid formulation approved for storage at 2 to 8 °C, while Genexol-PM^®^ is commercially available as a lyophilized powder [200]. Nanoxel^TM^ has been approved by the Drug Controller General of India since 2006, for the treatment of metastatic breast cancer, NSCLC, and AIDS-related Kaposi Sarcoma patients [202]. Paclical^®^, in certain countries Apealea^®^, is a CremophorEL-free paclitaxel formulation based on a XR17 micelle platform technology. It received market authorization from the EMA in November 2018 (EMA/791927/2018) to treat women with ovarian cancer.

#### 4.2.2. Dendrimers

Dendrimers (Figure 7) are hyperbranched three-dimensional polymeric nanostructures with functional moieties in the cavities and at the surface [166]. Polyester dendrimers are termed “smart carriers” for drug delivery applications, as they can be tailored for the complete release of their payloads in a specific environment, reducing the side-effects [203].

Dendrimers can be used for transdermal drug delivery as a substitute route of administration due to the reported unpleasant feedback when taken in oral dosage forms and for nauseated and unconscious patients [166]. Dendrimer uptake was analyzed 24 h after intravenous administration in rabbits, and less than 5% of the injected dose remained in circulation, with over 90% cleared out. G4-OH dendrimers are 4 nm in size and are expected to clear out via the kidney. In this model, dendrimers were not seen in the glomerulus 24 h after administration [161]. The use of ruthenium-terminated carbosilane dendrimers (CRD) significantly decreased the viability of pediatric leukemia cells (1301) with low toxicity for non-cancer cells (peripheral blood mononuclear cells—PBMCs) [204]. Moreover, Chittasupho et al. [205] formulated a CXCR4-targeted PAMAM dendrimer that decreased the migration and viability of an established B-cell-precursor-leukemia cell line derived from an adolescent male (NALM-6).

### 4.3. Inorganic Nanoparticles

Inorganic nanoparticles encompass metal nanoparticles (iron, gold, silver, and zinc) or rare-earth metal nanoparticles (lanthanum oxide, La_2_O_3_ or ytterbium oxide, Yb_2_O_3_) and silica nanoparticles, among others [20]. They have been widely used to diagnose and treat atherosclerosis or cancer [20]. The FDA has approved some inorganic nanoparticles intended for iron replacement therapies or for treating anemia and associated diseases (Table 6). Among them, Venofer^®^ and Ferrlecit^®^ have been studied for pediatric interventions. Venofer^®^ is an iron oxide nanoparticle coated with sucrose used for the slow dissolution of iron following intravenous injection, preventing a rapid and toxic increase in free iron in the blood. Ferrlecit^®^ is a stable macromolecular complex of sodium ferric gluconate in sucrose [161].

**Figure 9:**
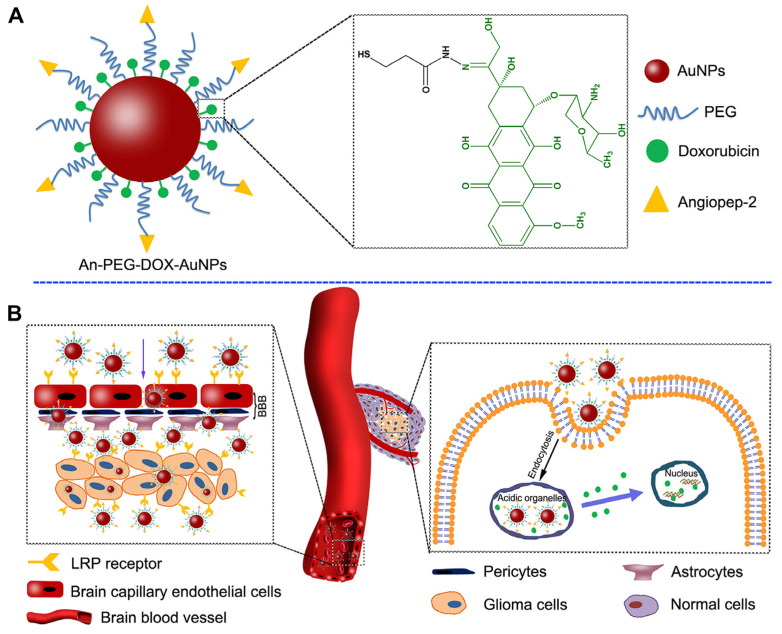
(**A**) Schematic representation and (**B**) delivery procedure of the angiopep-2-PEG-doxorubicin-gold nanoparticles (An-PEG-DOX-AuNPs). Briefly, the LRP1 receptor could mediate An-PEG-DOX-AuNP penetration through the BBB and targeting to glioma cells, after which DOX would be released at the tumor site or in tumor cells and enter into nuclei to induce tumor cell apoptosis. Reprinted from [207], copyright (2014), with permission from Elsevier.

Ongoing research in this field has highlighted the possible application of inorganic nanoparticles in diagnosing, treating, and monitoring pediatric brain tumors [206] and other pathologies [130]. Moreover, the application of hybrid nanoparticles has also revealed promising features [207]. For example, the use of Angiopep-2 (An)-PEG-doxorubicin (DOX)-gold nanoparticles (AuNPs) could penetrate the BBB and target glioma cells (Figure 9) [207].

### 4.4. Challenges in Using Nanotherapy in Pediatrics

As reviewed by us previously, the bright side of the coin in the application of nanotechnology in medicine may obscure dark shadows and it should further evolve as an auxiliary to circumvent troubleshooting in nanomedicine [20]. These challenges may impact not only the adult population, but particularly the pediatric population, as limited information for this age group is available [20,68]. Moreover, most preclinical studies to assess the impact of the physicochemical properties of nanosystems are conducted in adult models after intravenous administration, while the preferential route of administration for pediatrics is p.o. [208]. Additionally, the evaluation of the PK parameters of the nanoformulations could also be hindered, as reviewed elsewhere [161]. Other issues regarding the application of nanotherapies in pediatrics are transversal to those present for different dosage forms. However, here it is more evident because the topic of nanomedicine is more recent, and there is a vast unknown to explore [209].

**Figure 10:**
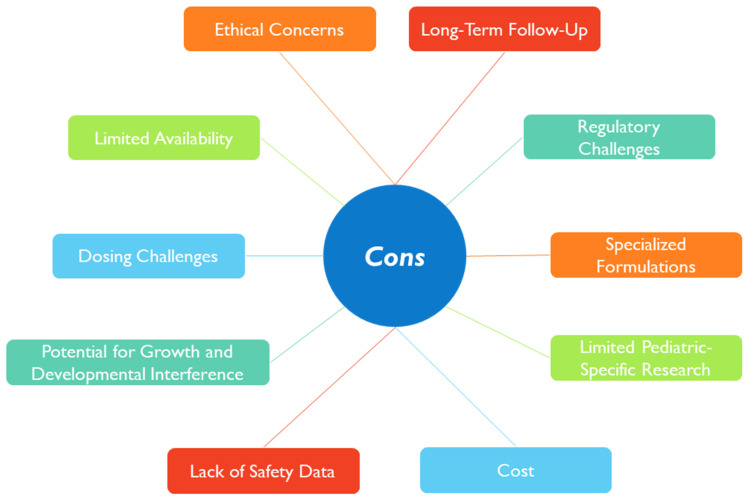
Summary of some issues that remain in developing nanotherapies for pediatric patients.

In Figure 10, a snapshot of the main issues that remain to be overcome in using nanotherapies in the pediatric age is presented.

When designing a nanomedicine intended for pediatric application, it would be beneficial to consider some of these points, particularly regarding the safety and efficacy that could contribute to long-term effects [210]. It would also be relevant to study how environmental exposure to nanoparticles could impact children’s health, development, and their treatment response [211].

Moreover, ethical concerns regarding informed consent in this age group for enrollment in clinical trials, the lack of public understanding of nanotechnology, and socioeconomic issues may also limit the studies using nanoparticles in children [130]. The pros and cons of nanomedicine should cross all stages during the nanomedicine design and development, focusing on the well-being and the best interest of children.

Taking into account potential benefits, nanomedicine has been dubbed, together with ATMPs, as the “therapies for the future” by the European Parliament [23]. The following section summarizes some advancements and issues of ATMPs, mainly focusing on the pediatric population.

## 5. Advanced Therapy Medicinal Products (ATMPs) for Pediatric Healthcare

**Figure 11:**
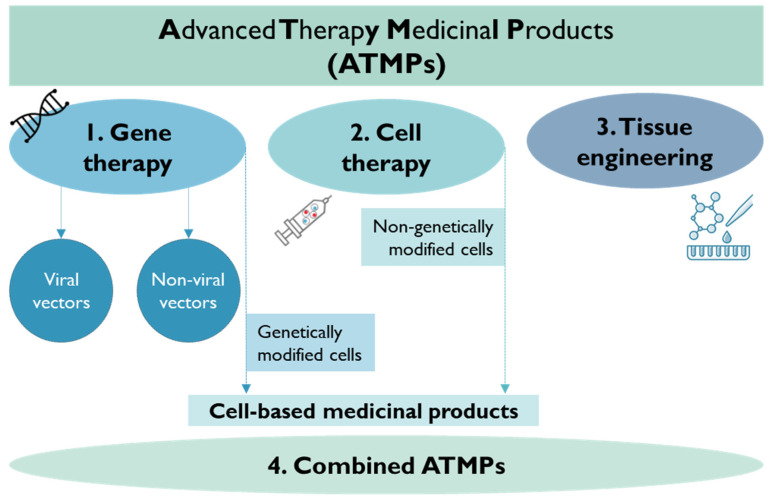
Schematic summary of the current available advanced therapy medicinal products (ATMPs).

ATMPs are medicinal products that encompass (1) gene therapy medicinal products (GTMPs), (2) somatic cell therapy medicinal products (sCTMPs), (3) tissue-engineered products (TEPs), and (4) combined ATMPs (e.g., tissue or cell-associated with a device) (Figure 11) [212,213]. The decision dendrogram regarding the different types of ATMPs can be found in the EMA reflection paper: EMA/CAT/600280/2010 rev.1.

### 5.1. ATMPs—Legal Framework in the European Union

The EMA is the agency that regulates the free movement of ATMPs within the EU, to facilitate market access to these medicines, foster the competitiveness of European pharmaceutical companies, and ensure health protection for patients. The EMA’s Innovation Task Force (ITF) arose to promote the development of effective, innovative medicines that could be available to patients promptly. Based on this, the EMA has encouraged the development of ATMPS by offering advisory services and incentives.

As for all medicinal products, to obtain marketing authorization, the development of ATMPs should follow the requirements of good manufacturing practice (GMP) stated in the Commission Directive 2003/94/EC, with a specific focus on the GMP guidelines that mainly address ATMPs, presented in the “Good Manufacturing Practice for Advanced Therapy Medicinal Products” (C(2017) 7694 final guideline, from Brussels 2017-11-22). Some important EU GMP guidelines could also be of interest for ATMPs manufacturing, such as Annex 2 of the “Manufacture of biological active substances and medicinal products for human use”, Annex 13 of the “Manufacture of investigational medicinal products”, and Annex 16 of the “Certification by a qualified person and batch release”. Moreover, good clinical practice (GCP) requirements should also be applied for ATMPs, which are described in the Commission Directive 2005/28/EC with complementary details specific for ATMPs in the EC guideline C(2019) 7140 final (Brussels, 2019-10-10). Aligned with these, good laboratory practice (GLP) procedures also need to be taken into consideration concerning ATMPs [214].

Furthermore, in February 2018, the EMA released a draft of the revised guidelines on safety and efficacy follow-up and risk management of ATMPs, EMEA/149995/2008 rev.1. The guideline describes specific aspects of pharmacovigilance, risk management planning, safety, and efficacy follow-up of authorized ATMPs, as well as some elements of clinical follow-up of patients treated with ATMPs.

The overall regulation of ATMPs is summarized in Regulation (EC) No 1394/2007, with a distinct reference to the Committee for Advanced Therapies (CAT) that ensures the trinomial of quality, safety, and efficacy of ATMPs, and provides an up-to-date overview of the scientific landscape in the field. The CAT is also responsible for providing recommendations and scientific advice on classifying ATMPs (Article 17 of Regulation (EC) No. 1394/2007). Micro-, small- and medium-sized companies can also submit requests for certification to the CAT under the Article 18 of Regulation (EC) No 1394/2007, corresponding to the ATMPs regulation.

**Figure 12:**
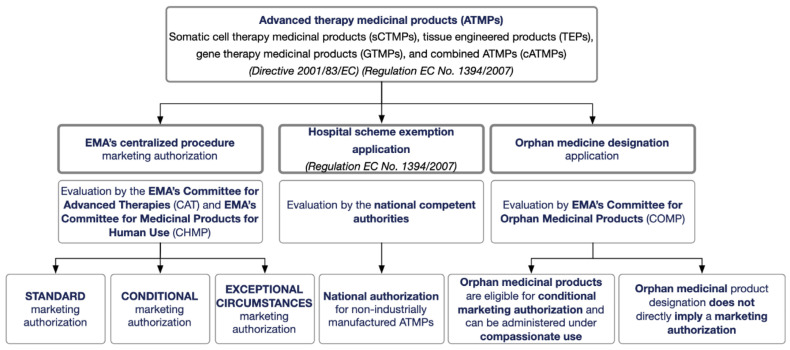
Regulatory framework followed by EMA for marketing authorization of ATMPs in the European Union. Reprinted from [212], under a CC BY 4.0 license.

The Marketing Authorization Application (MAA) procedure for ATMPs requires their evaluation based on the centralized procedure, described in the Regulation (EC) No 726/2004, with the preliminary assessment from the CAT, which deals with the classification of the ATMPs [215]. The centralized procedure can encompass three types of marketing authorization (MA): standard marketing authorization, conditional marketing authorization, and marketing authorization under exceptional circumstances (Figure 12) [216]. A typical MA is conferred when no additional information on quality, safety, and efficacy or in the benefit–risk balance of the medicinal product under evaluation is required regarding that presented in the MAA. A conditional MA may be applied when an unmet medical need supports the availability of medicine to patients before the comprehensive clinical data. An MA attributed in exceptional circumstances occurs only in extreme situations, like when a disease is rare or a clinical endpoint is difficult to measure, and the safety and efficacy data required for a standard MA are pretty challenging to obtain based on the limited data originated from the reduced number of patients [215].

The centralized procedure is characterized by a single application, evaluation, and authorization through all the EU member states, including the European Free Trade Association (EFTA) members, such as Iceland, Liechtenstein, and Norway. In the case of medicines derived from biotechnology processes, orphan medicinal products, and medicines aiming to treat diseases such as cancer or HIV, the centralized procedure is compulsory [215,216]. On the other hand, it could be optional in cases when the new active substances provide other indications than those stated previously, or for those that present scientific, therapeutic, and technical innovation, or whose authorization is considered of particular relevance for the public and animal health in the EU [215,216].

In the centralized procedure, after receiving the application from the developers of the ATMPs, the CAT prepares a draft opinion about the quality, safety, and efficacy of the ATMPs received [216]. Based on the CAT opinion report, the CHMP adopts an opinion recommending (or not) the authorization of the ATMP to the European Commission, which is responsible for the final decision.

Interestingly, the so-called “hospital scheme exemption application” under Regulation EC No 1394/2007 launched the opportunity for a national authorization of non-industrially manufactured ATMPs. Based on this, if an ATMP is designed and produced for an individual patient, it can be used on a non-routine basis in a hospital under the exclusive responsibility of a specific medical practitioner [216].

An ATMP can also be classified as an orphan medical product by the Committee for Orphan Medicinal Products (COMP) of the EMA [216]. The orphan designation is attributed if the disease for which it is intended is a high-risk or chronically debilitating disease, it does not affect more than 5 in every 10,000 people, and there is no other satisfactory therapy. Therefore, it fills a gap and offers benefits to patients. The orphan designation procedure was implemented by the EMA in 2000 with Regulation (EC) No 141/2000 together with the amended Regulation (EC) No 847/2000 that provides definitions and rules for implementation [215]. More guidance could also be found in the 2016/C 424/03. More recently, the EMA released guidance on the designation of ultra-rare disease (Regulation (EU) No 536/2014). The EMA launched the PRIME initiative to accelerate the process of bringing medicines to market, which allows increasing support in developing treatments for unmet medical needs [217]. This scheme enforces communication between the applicant and the EMA from the earliest stages of drug development, which facilitates access to incentives to generate robust data on efficacy and safety for timely access evaluation at the time of application, culminating in the faster arrival of the new medicine to patients [215]. There could also be benefits from marketing exclusivity if designated as an orphan medicinal product. Additionally, even if a product is similar to the one approved, an MA can still be granted for the second product if it is safer, more effective, or otherwise clinically superior [215]. Due to the signs of progress in the development of innovative therapies, particularly in ATMPs, the definition of the concept of a similar medicinal product evolved and on 29 May 2018, the EC Regulation (EU) 2018/781 amended Regulation (EC) No. 847/2000 [215].

Regarding the MAA of ATMPs for the pediatric age, they faced the same routes of application as in adults [25], with crucial regulatory support provided in the Pediatric Regulation, Regulation (EC) No 1901/2006 and its amendment (EC) No 1902/2006. Recognizing the shortfall of pediatric treatments, the Pediatric Regulation offers incentives such as those stated in the pediatric-use marketing authorization (PUMA), access to the EMA-specific pediatric expert committee, and free advice to the industry. In line with this and to further promote the dissemination of pediatric trial results, clinical trials for pediatric interventions in the EU are entirely covered by the EU Clinical Trials Register [25].

Additionally, ATMPs’ post-authorization is guided by the EMA good pharmacovigilance practices (GVP), regarding the draft “Guideline on safety and efficacy follow-up and risk management of Advanced Therapy Medicinal Products” (EMEA/149995/2008 rev.1, 2018) that focuses on the single characteristics of ATMPs in line with Article 14 (4) of the Regulation (EC) No 1394/2007. It also offers a framework for the early mitigation of risks and their consequences to the patients, focusing on the post-authorization follow-up on the safety and efficacy of ATMPs.

The complexity and uniqueness of ATMPs, aligned with their intrinsic heterogeneity, brings some issues in the regulatory strategies, including the need for specialized and certified centers that could help in developing ATMPs, an active framework of follow-up, accessibility and financial and sustainable portfolios, access to robust clinical trials, and the development of animal models that better fit the human profile, all with the trinomial of quality, safety, and efficacy as the main pillars [218].

### 5.2. FDA and EMA-Approved ATMPs in Pediatrics

Most EMA-approved ATMPs are not indicated for pediatric patients (EMA/CAT/50775/2023). A similar profile has been registered in the US by the FDA [219]. Table 7 summarizes the EMA and the FDA-approved ATMPs indicated for the pediatric age.

Most ATMPs currently on the market are GTMPs aimed at treating rare diseases. Interestingly, all the EMA-approved ATMPs listed in Table 7 received orphan medicinal product status and were approved by the PRIority MEdicines (PRIME) scheme.

The following section shall propose key developments using ATMPs particularly targeting the pediatric population [25,220,221,222,223].

### 5.3. Gene Therapy

Gene therapy medicines relate to applying recombinant nucleic acids to treat, prevent, or cure a disease or medical disorder [224].

Gene therapy can be based on three main strategies: ex vivo, in vivo, or in situ [225]. Ex vivo gene therapy involves the genetic modification of cells outside the body to produce therapeutic factors and their subsequent transplantation into patients [226]. Unlike ex vivo therapy, in vivo gene therapy aims to modify the genetic repertory of target cells within living organisms [227].

The development of safer and more efficient viral vectors based on retro and lentiviruses, combined with improved technology for the scalable production of viral vectors, has enabled the successful therapy of rare genetic disorders [228]. In 2016, the first ex vivo gene therapy worldwide, Strimvelis^TM^, based on hematopoietic stem cells (HSC), was approved by the EMA for the treatment of severe combined immunodeficiency caused by adenosine deaminase deficiency (ADA-SCID) in pediatric patients that did not have an adequate cell donor [229]. A single infusion of autologous bone-marrow HSC, gene-corrected by γ-retrovirus-based technology, resulted in the long-term correction of T lymphocyte activity, immune reconstitution, and 100% survival during a 7-year follow-up. Additionally, the lack of leukemic transformation in transduced cell clones provides evidence of safety as well as hope for the successful approval of this type of gene therapy for other genetic diseases. Similar clinical benefits were observed in patients with Wiskott–Aldrich syndrome [230,231], X-linked adrenoleukodystrophy, metachromatic leukodystrophy [232,233], or transfusion dependent β-thalassemia [234,235,236] treated with lentiviral (LV) HSC therapy. In the case of X-linked severe combined immunodeficiency, LV therapy proved successful in both pediatric and adolescent patients suffering from secondary effects of previous allogeneic HSC transplant [237]. The use of such autologous therapy also circumvents the limitations of allogenic therapies by evading host–recipient immunologic differences and the need for severe immune suppression. Nonetheless, numerous factors can influence the therapeutic outcome in individual patients or different diseases, and they have been described in detail in a recent review by Naldini [228]. Additionally, the implementation of ex vivo therapy is limited by the requirements of highly specialized experts involved in all stages of the product production and performance, the short shelf life of genetically altered cells, and high costs. In order to fully exploit the therapeutic potential of ex vivo gene therapy, the long-term monitoring of risks related to insertional mutagenesis and oncogenesis, immunogenicity, and off-target effects is needed.

Alipogene tiparvovec (Glybera^®^) was the first approved gene therapy [222]. In 2022, the EMA approved Upstaza^TM^, a gene therapy medicine based on eladocagene exuparvovec, a functional gene within the adeno-associated viral vector, for use in children aged >18 months with severe aromatic L-amino acid decarboxylase (AADC) deficiency (EMA/365735/2022).

Nonetheless, despite the progress in viral vector development, some issues related to immunogenicity, low loading capacity, and difficulty in large-scale production are still limiting their translation into clinical practice and have inspired the investigation of alternative, potentially more successful and safer delivery vectors [238]. Therefore, to overcome challenges related to viral vectors, non-viral vectors based on cationic lipids or polymers have been pursued [238].

Examples of cationic lipids that are commercially available are 1,2-dioleoyl-3-trimethylammoniumpropane (DOTAP), N-[1-(2,3-dioleyloxy)propyl]-N,N,N-trimethyl-ammonium chloride (DOTMA), 2,3-dioleyloxy-N-[2(sperminecarboxamido)ethyl]-N,N-dimethyl-1-propanaminium trifluoroacetate (DOSPA), and 1,2-dimyristyloxypropyl-3-dimethyl-hydroxyethyl ammonium bromide (DMRIE). Cationic liposomes have emerged as attractive gene vectors because they enhance pharmacokinetic properties and present relatively low immunogenicity [239]. However, the drawbacks of cationic lipid-based nanocarriers, such as poor stability, low transfection efficacy, and the generation of inflammatory responses, have limited their further application [239].

One of the most studied non-viral vectors for nucleic acid delivery is polyethyleneimine (PEI), which has been considered the gold standard since 1995. PEIs are a group of synthetic, water-soluble, linear, or branched polymers (Figure 8) composed of primary, secondary and tertiary amine groups that confer positive charge density at physiologic pH. Moreover, the “proton-sponge” effect makes them suitable for gene therapy, protecting the nucleic acid cargo from lysosomal degradation [238]. Currently, only one clinical trial is recruiting to test a PEI-based vaccine. The early phase I clinical trial, NCT04049864, aims to evaluate the safety and immunogenicity of a vaccine composed of DNA conjugated with a linear PEI (20 kDa) targeting relapsed neuroblastoma patients with ages between 1 and 20 years old (ClinicalTrials.gov database, accessed on 9 August 2023). The combined form of the vaccine includes an intramuscular injection of the DNA-PEI conjugate (polyplex) and oral administration using the attenuated *Salmonella enterica* as DNA vaccine carriers. The direct correlation between high molecular weight and high positive charge density may explain the scarcity of clinical data using PEI. While it is advantageous for high transfection efficiency, it may lead to undesirable, off-target toxicity [240]. Therefore, a balance between molecular weight and efficient transfection has been recommended, which has proved challenging. For this, PEI was grafted with hydrophobic moieties like lipoic acid, deoxycholic acid, cholesterol, or phospholipids to improve the transfection efficacy [238]. Furthermore, Wang et al. [241] developed hyperbranched-star PEG-g-PEI as a promising nonviral carrier for gene delivery in retinoblastoma, the most common malignant intraocular childhood tumor.

PEIs can also be conjugated with other polymers, such as Pluronics^®^, for gene and drug co-delivery [242]. This approach has also been tested for treating pediatric malignancies, such as osteosarcoma [243]. Despite being considered a high transfection non-viral vector, PEI is a non-biodegradable polymer that accumulates around the cell and triggers cytotoxicity, possibly hampering its translation to the clinic [244].

Poly(amidoamine) (PAMAM) are monodisperse and hyper-branched polymers (Figure 8), [245], that have been widely exploited for gene delivery. A major disadvantage of those common dendrimers is their toxicity, associated mainly with the chemistry of the surface amine groups. In a study performed by Wang et al. [246], the PAMAM dendrimer was modified with triazine-containing polymers as a strategy for efficient tumor necrosis factor (TNF)-related apoptosis-inducing ligand (TRAIL) gene therapy of osteosarcoma [246]. More recently, generation four of the PAMAM dendrimer has demonstrated potential for drug, peptide, and DNA delivery [247]. In spite of its high density of positive charges, which may contribute to its toxicity, in some cases, such as in cancer treatment, as the tumor cells present excessive intracellular negative charges, it could be selectively advantageous [247].

Poly(vinylimidazole) (PVI) (Figure 8) is a water-soluble polymer with a protonable imidazole group at acidic pHs [248]. PVI has additional biocompatibility properties, limited toxicity, and the ability to escape the endosome by activating the “proton sponge” mechanism [244]. The use of PVI for biomedical applications alone [249] or in combination with other polymers such as poly(acrylamide) [250] or chitosan [248] has already been exploited. Particularly, due to the presence of the imidazole group, PVI has been reported to present significant antibacterial activity [251,252].

Mumper et al. reported chitosan (Figure 8) as a potential gene carrier in the mid-1990s [253]. The pKa of amino groups on chitosan is around 6.5, so they tend to remain protonated at acidic and neutral pH [254]. Chitosan is positively charged and soluble in weakly acidic solutions, with a charge density dependent on the pH and the degree of deacetylation [255]. Ta et al. [256] formulated a chitosan hydrogel for pediatric osteosarcoma gene therapy using the pigment epithelium-derived factor (PEDF), with promising anti-tumor activity in vitro (SaOS-2 cells) and in vivo.

More recently, the Clustered Regularly Interspaced Short Palindromic Repeats)/CRISPR-associated protein 9 (CRISPR/Cas9) gene-editing technology has revolutionized gene therapy, as it can permanently correct deleterious base mutations or disrupt disease-causing genes with great precision and efficiency [257]. This technology can help reduce the risk of death in children under the age of five [258]. Therefore, its application to address infectious diseases in the pediatric population has been explored [258]. Malaria is a life-threatening infectious disease that children <5 years old are most vulnerable to, and it is transmitted through the bite of an infected Anopheles mosquito carrying the Plasmodium parasite. A CRISPR/Cas9-based gene editing approach based on the *Fibrinogen-Related Protein 1* (*FREP1*) gene knockout, a fundamental protein for the survival of Plasmodium, was described by Dong et al. [259] in their search for malaria treatment. Moreover, CRISPR-Cas9 technology has been studied for the treatment of severe monogenic diseases, such as transfusion-dependent β-thalassemia (TDT) and sickle cell disease (SCD), by targeting the *BCL11A* erythroid-specific enhancer, which is responsible for the repression of γ-globin expression and fetal hemoglobin in erythroid cells [260]. Based on this approach, there are two clinical trials, NCT03655678 and NCT03745287, enrolling children (>12 years old) to assess the safety and efficacy of autologous CRISPR-Cas9 Modified CD34^+^ Human Hematopoietic Stem and Progenitor Cells (hHSPCs) in subjects with TDT and SDS, respectively [260]. In another study, Webber et al. developed a CRISPR/Cas9 system to correct *COL7A1* gene [261], which causes recessive dystrophic epidermolysis bullosa (RDEB), a disease that affects the skin and other organs, in which children that are born with this condition are referred to as “butterfly child” [262].

#### 5.3.1. GTMPs—Guidelines on Quality, Pre-Clinical, and Clinical Aspects

Quality, safety, and efficacy requirements of GTMPs should be guaranteed from the beginning of their manufacturing. The EMA/CAT/GTWP/671639/2008 (2020) is a revised version of the “Note for Guidance on the Quality, Preclinical and Clinical aspects of gene transfer medicinal products” (CPMP/BWP/3088/99), published in 2001, that aims to guide the development and evaluation of GTMPs intended for human use and presented for MAA. Moreover, the EMA/CAT/80183/2014 (2018) offers guidance on quality, non-clinical and clinical aspects of gene therapy medicinal products.

One of the parameters of the utmost importance for the quality of GMTPs is the origin of the vector, which can be of viral, bacterial or non-viral nature. Some considerations that should be taken into consideration are genotypic and phenotypic characteristics, identity, purity, potency, biological activity of the therapeutic sequence, transduction efficiency, mechanism of action, and their relation to the specificity of GTMP (EMA/CAT/80183/2014). Vectors should be produced from well-characterized bacterial or virus seeds and/or cell banks. For bacterial vectors, it is required to describe the isolation, nucleotide sequences, functions and main characteristics of the bacteria’s genome. Although the full sequencing is not required, manipulated regions should be described in detail (EMA/CAT/80183/2014; EMA/CAT/GTWP/671639/2008). For viral vectors, tropism, the ability to infect cells, virulence, replication capacity, the proportion of infectious to non-infectious particles, immunological characteristics, the average size of particles and aggregates, and the insertion sites determined along with the insertion potential and associated risks are required (EMA/CAT/80183/2014; EMA/CAT/GTWP/671639/2008). For RNA and DNA plasmid vectors, aspects such as the characterization of identity, genetic integrity, the absence of foreign agents, sterility, sequence confirmation, and the presence/absence of specific characteristics such as CpG sequences should be documented (EMA/CAT/GTWP/671639/2008). Moreover, when a nucleic acid vector is associated with a nanoparticle, the characteristics of the vector, complex components, and nucleic acid sequence must be investigated, along with the structure of the complex and the interaction between the carriers and the negatively charged DNA (EMA/CAT/80183/2014).

The EMA/CAT/80183/2014 proposed some tests that are expected to be included in the set of specifications (see ICH guideline Q6B, Ph.Eur. 5.14 and Ph. Eur. 2.6.16). These include (1) identity and integrity, (2) content, (3) potency assay, (4) product related impurities, (5) process-related impurities, and (6) pharmacopeial tests.

When screening for the virulence of viral vectors, the use of an assay of suitable sensitivity is of paramount importance (EMA/CAT/80183/2014; EMEA/CHMP/ICH/449035/2009), as is ensuring that the risk of microbiological contamination during the manufacturing process is minimized. In some cases, it may be acceptable to release Replication Competent Viruses (RCV) within the limits imposed by the non-clinical and/or clinical evidence (EMA/CAT/GTWP/671639/2008). Tests for retroviruses include assays to assess the ability to infect sensitive cell cultures and electron microscopy studies. If the ability to infect is not detected and no retroviruses or retrovirus-like particles are observed using electron microscopy or reverse transcriptase, other appropriate assays should be performed to detect retroviruses that may be non-infectious (CPMP/ICH/295/95). In some cases, particularly when using lentiviruses or retroviruses, the risk of integrating the germline is increased (CHMP/ICH/469991/2006). To address this issue, the most frequently used test is the quantitative PCR.

### 5.4. Cell Therapy

Cell therapy spans multiple therapeutic areas, such as regenerative medicine, immunotherapy, and cancer therapy. It combines stem- and non-stem-cell-based unicellular or multicellular therapies, typically employing autologous or allogeneic cells, administered topically or as injectables, infusions, bioscaffolds, or scaffold-free systems [220]. The global cell therapy market size is estimated to achieve a CAGR of ca. 17% from 2023 to 2030 [263,264]. Although cell-based therapies present some safety concerns regarding potential tumorigenicity and high manufacturing costs, they have unique intrinsic features that offer the potential for enhanced efficacy against disease [221].

Stem cell therapies can be grouped into three categories: pluripotent stem cells (PSCs), adult stem cells (ASCs), and cancer stem cells (CSCs) [220]. Currently, the use of PSC- and ASC-derived organoids is considered a hot topic in translational stem cell research, as they can offer the three-dimensional (3D) structural and functional mimicry of organs in vitro [265]. Generally, the clinical use of CSCs has been motivated by their capacity to interfere with multiple signaling pathways, preventing cancer growth and relapse [266]. Across the world, stem and progenitor cell therapy is based on hematopoietic or mesenchymal cells, and is currently approved for various types of blood cancers, various blood disorders or tissue regeneration (Table 7) [267,268]. Cell-based therapies for humans primarily focused on bone marrow transplants for patients with blood-borne cancers in the middle of the XX century, and have resulted in a variety of currently FDA-approved products [221]. For example, allogeneic stem cell transplant therapy based on hematopoietic stem cells originating from umbilical cord blood (Omisirge^®^) was approved in 2023 by the FDA for use in patients above 12 years of age for the treatment of hematologic malignancies, while other therapies remain experimental in the USA.

A recent survey of ClinicalTrials.gov and the World Health Organization (WHO) International Clinical Trials Registry Platform (ICTRP) revealed 202 clinical studies related to the implementation of stem cell therapies for pediatric diseases [269]. Although the number of studies has tended to increase since 2007, the majority of the 112 completed studies) were short-term (<36 months), single-center clinical studies with a low number of recruited patients (<50) and without gender restrictions. Only about 30% of the studies with primary completion published results. While the studies were mostly based on HSC and mesenchymal stem cells, in the past 5 years the emphasis was mostly on allogeneic transplants. The low power of trials may obscure both clinically relevant results and adverse effects, stressing the need for larger multi-center studies in order to confirm clinical applicability and avoid experimental bias [269].

On the other hand, the management of non-stem-cell-based therapies has indicated the use of somatic cells that are isolated from the human body, propagated, expanded, selected, and subsequently administered to patients for curative, preventive, or diagnostic purposes [220]. Non-stem-cell-based cell therapies include fibroblasts, chondrocytes, keratinocytes, hepatocytes, pancreatic islet cells, and immune cells, such as T cells, dendritic cells (DCs), natural killer (NK) cells, or macrophages [220].

#### 5.4.1. Chimeric Antigen Receptor T Cell Therapy

CAR T cell therapy is an example of a cell-based gene therapy. This type of treatment combines gene and cell therapy [270]. Its regulatory approval by the FDA has revolutionized this clinical research area [221]. In CAR T cell therapy, immune cells are removed from a patient, genetically modified, expanded and cryopreserved under GMP, and then placed back into the original patient to fight against cancer [271,272]. This process is usually performed manually or in a semi-automated manner, leading to variability and high costs and significantly impacting the time to use. The pharmaceutical sector and academic institutions have accomplished prompt efforts to reduce the manufacturing time from 14 days to a few days or even one day. The manufacturing processes of CAR T cells use quality controls (QCs) to monitor the production process and to approve the final product for application in patients. Currently, as the most common production system is the all-in-one bioreactor, the recommended QCs are gas, temperature, and pH value. Regarding the molecular and cellular QCs, the most commonly used are cell viability, cell number, cell identity, purity, or CAR receptor expression, which are manually acquired and processed, leading to delays in application and limiting the real-time edition of the production process [272]. Therefore, integrating automated manufacturing workflows with on-line or in-line monitoring of the production process, for example by developing microfluidic qRT-PCR or flow cytometry devices connected to bioreactor platforms, could be of interest to achieve optimized QCs.

Moreover, the use of label-free biophysical methods, such as real-time cell deformability (mechanical) cytometry or autofluorescence imaging, could contribute to improving QC processes, taking advantage of real-time analysis in a minimally invasive manner [272]. Furthermore, machine-to-machine communication and efficient data processing systems, like digital twins, may contribute to the optimization of the production processes of CAR T cells in real-time (or close to that) [272]. Therefore, optimizing protocols for biological research, process development, and hardware technologies could contribute to the automatic production of autologous CAR T cells, considering regulatory compliance and personalized treatment approaches [272].

CD19-CAR T cell therapy has been a medical breakthrough in treating pediatric ALL, as reviewed elsewhere [271]. Nonetheless, some adverse effects are associated with CAR T therapy. Acute life-threatening complications such as cytokine release syndrome (CRS) and immune effector cell-associated neurotoxicity syndrome (ICANS) have been observed, as well as side effects caused by profound B-cell aplasia that require human IgG substitution to prevent severe infectious complications [273]. Additionally, limited or lacking efficacy has led to the need to develop advanced CAR technologies [274]. In the majority of cases, the *CAR* gene is delivered into cells by viral vectors. Alternatively, the use of non-viral vectors such as mRNA technologies, transposons, or nanoparticles could improve their longevity and safety [275]. To that end, innovative engineering approaches such as genome and epigenome editing, synthetic biology, and biomaterials are being exploited [221,276].

### 5.5. Tissue-Engineered Products

Tissue-engineered products contain or consist of engineered cells or tissues, and display properties when they are administered to human beings that allow them to regenerate, repair, or replace human tissue (EC No. 1394/2007). Cells or tissues are considered engineered if they fulfill at least one of the following conditions: (1) have been subjected to substantial manipulation or (2) are not intended to be used for the same essential function or functions in the recipient as in the donor [212].

Deguchi et al. [277] recently reviewed the use of tissue-engineered products with relevant applications to pediatric surgery.

### 5.6. Combined ATMPs

Combined ATMPs (cATMPs) are composed of a GTMP, sCTMP, or TEP in combination with one or more medical devices or one or more active implantable medical devices as an integral part of the product (EMA, EC No. 1394/2007), which may include devices such as biomaterial cell scaffolds or nanoparticles for gene therapy delivery [278].

cATMPs represent only 1% of the ATMPs that are under development in the EU [212]. Following these regulations (Directive 93/42/EEC and Directive 90/385/EEC) and the MEDical DEVices guidance document (MEDDEV), a medical device must be approved with the CE marking, an abbreviation in French of “Conformité Européenne” (European Conformity), prior to commercial availability in the EU. In this regard, any medical device that includes a cATMP must be previously approved with the CE marking by the notified bodies for its commercialization in the EU [212]. Moreover, when the final product includes a medical device, specific release tests may be required (EMA/CAT/80183/2014).

Wilkins et al. have recently presented a pipeline for ATMPs, demonstrating that combined ATMPs possess great interest in cardiovascular system diseases, along with ophthalmology/endocrine/nutritional/metabolic/genetic disorders and hematological malignancies [279]. Besides that, no clinical trial seems to be registered in the EU database using cATMP for neurological applications [278].

In 2013, the only cATMP approved in the EU was for the repair of knee cartilage defects. However, it was withdrawn in 2014 for commercial reasons due to the closure of the EU manufacturing site [212]. Later, in 2017, Spherox was recommended for marketing authorization to repair cartilage knee defects in adult populations (EMA/CHMP/315817/2017).

The current lack of combined ATMP approaches on the market and in clinical trials may represent an important research and investment opportunity [278].

## 6. Future Perspectives and Final Remarks

The pediatric drug development landscape has undergone significant changes in recent decades in moving towards more efficient and safer therapeutics, but some issues remain unaddressed. Factors that can impact the pediatric pharmacotherapy practice and drug development are (1) a lack of approved APIs for the pediatric population, (2) a deficiency in regulatory clarity, (3) low market size and profitability, (4) age-appropriate drug formulations, (5) a lack of safety data for excipients used in pediatric drug development, (6) the route of administration not being age-adjusted, (7) complete pharmacokinetic data not being available, and/or (8) difficulties in establishing in vivo models that can mimic different pediatric subgroups, leading to the need for novel technologic and galenic requirements. Using nanomedicine seems to provide a way to overcome some of the reported issues. Preclinical and clinical studies offer promising results in improving the solubility, organoleptic properties, therapeutic efficiency, and safety of a broad spectrum of APIs. However, a considerable rift needs to be crossed until most of the bench-formulated nanomedicines can be translated to the patient’s bedside, particularly in the case of pediatric nanomedicines. A workforce has been proposed that joins pharmaceutical developers and physicians in standardizing procedures for the development of pediatric formulations. The advent of ATMPs brings the possibility of curing pediatric pathologies with complete remission of the disease. However, challenging questions regarding their safety and immunogenic adverse effects persist. These innovative therapies also provide challenges for healthcare systems and drug developers, summarized in the “four As”: authorization, availability, assessment, and affordability.

Moreover, pharmacovigilance issues may also hamper the number of ATMPs currently available in clinical practice. Furthermore, economic problems due to the high costs necessary for the development of these technologies, as well as the limited revenue, may also impair investment in this area. Aligned with these, a call for action emitted by the Alliance for Regenerative Medicine (ARM) revealed that the EU is becoming stagnant compared to the U.S. and Asia in the number of therapeutic developers, clinical trials, and investments nurturing the development of ATMPs.

Therefore, some so-far-unanswered questions have arisen: (1) does pediatric medicine continue to be a “therapeutic orphan?” (2) Are the healthcare and the economic systems prepared for a personalized medicine perspective? (3) Are governments, the regulatory entities, and society prepared for the technophilic and technophobic demands proposed by the nanomedicine advancements, particularly employing intelligent nanomaterials, and those arising from ATMPs?

Pediatric medicine continues to be a hot and challenging topic to be investigated and the role of pharmaceutical developers is undoubtedly crucial in the pre-conception, design, and formulation, to the clinical phase of them, taking into consideration a common international framework established in the trinomial pillars of quality, efficacy, and safety in a fit-by-design perspective. 

## Figures and Tables

**Table 1:** Summary of Food and Drug Administration (FDA)-approved lipid-based nanoformulations.

Commercial Name	Active Agent	Composition (Molar Ratio)	Indication	Approval Year	Ref.
Cancer					
Doxil^®^	Doxorubicin	HSPC:Cholesterol:PEG 2000-DSPE (56:39:5)	Kaposi’s sarcoma, ovarian cancer, multiple myeloma	1995	[146]
DaunoXome^®^	Daunorubicin	DSPC:Cholesterol (10:5)	Kaposi’s sarcoma	1996	[147,148]
DepoCyt^®^	Cytarabin and AraC	Cholesterol:Triolein:DOPC:DPPG (11:1:7:1)	Complication of lymphoma lymphomatous meningitis	1999	[149]
Myocet^®^	Doxorubicin	EPC:Cholesterol (55:45 molar ratio)	Metastatic breast cancer	2000	[150]
Mepact^®^	Mifamurtide	DOPS:POPC (3:7)	Osteosarcoma	2004	[150]
Marqibo^®^	Vincristin	Sphingomyelin:Cholesterol (60:40)	Acute lymphoblastic leukemia	2012	[149]
Onivyde^TM^	Irinotecan		Metastatic pancreatic adenocarcinoma	2015	[150]
Vyxeos^®^	1:5 molar ratio of daunorubicin:cytarabine	DSPC:DSPG:Cholesterol (7:2:1)	Acute myeloid leukemia	2017	[151]
Infection					
Arikayce^®^	Amikacin	DPPC:Cholesterol (2:1 weight ratio)	Pulmonary infection caused by *Mycobacterium avium*	2018	[152,153]
Abelcet^®^	Amphotericin B	DMPC:DMPG (7:3)	Fungal infections	1995	[150]
Amphotec^®^	Amphotericin B	Cholesteryl sulphate:Amphotericin B (1:1 molar ratio)	Fungal infections	1996	[150]
AmBisome^®^	Amphotericin B	HSPC:DSPG:Cholesterol:Amphotericin B (2:0.8:1:0.4)	Fungal/protozoal infections	1997	[154]
Pain management					
DepoDur^TM^	Morphine sulfate	DOPC, DPPG, Cholesterol, Triolein	Pain management	2004	[150]
Exparel^®^	Bupivacaine	DEPC, DPPG, Cholesterol and Tricaprylin	Pain management	2011	[150]
Photodynamic therapy (Ophtalmic)					
Visudyne^®^	Verteporfin (Photosensitizer)	Verteporphin:EPG:DMPC (1:3:5)	Wet age-related macular degeneration, myopia, ocular histoplasmosis	2000	[149,155]
Nucleic acid therapy					
Onpattro^TM^ (Patisiran)	siRNA lipid formulation designed to target transthyretin (TTR) mRNA in the liver cells		Hereditary transthyretin amyloidosis (hATTR)	2018	[156,157,158]
Vaccines					
Epaxal^®^	Inactivated hepatitis A virus (strain RGSB)	DOPC:DOPE (75:25)	Hepatitis A	1993	[150]
Inflexal^®^V	Inactivated hemaglutinine of Influenza virus strains A and B	DOPC:DOPE (75:25)	Influenza	1997	[150]
mRNA-1273	mRNA	Positively charged lipid:PEGylated lipid:Cholesterol:DSPC (50:1.5:38.5:10)	COVID-19	2020, Emergency Use Authorization	[159,160]
BNT162b2	mRNA	Positively charged lipid:PEGylated lipid:Cholesterol:DSPC (46.3:1.6:42.7:9.4)	COVID-19	2020, Emergency Use Authorization	[159,160]

Abbreviation: hydrogenated soy phosphatidylcholine (HSPC); PEG2000-DSPE poly (ethylene glycol)-distearoylphosphatidylethanolamine (PEG2000-DSPE); dimyristoylPEGphosphatidylcholine (DMPC); dimyristoylphosphatidylglycerol (DMPG); distearoylphosphatidylcholine (DSPC); distearoylphosphatidylglycerol (DSPG); egg phosphatidylglycerol (EPG); egg phosphatidylcholine (EPC); dioleoylphosphatidylcholine (DOPC); dipalmitoylphosphatidylglycerol (DPPG); distearoylphosphatidylcholine (DSPC); [3-[3-(2-methoxyethoxy)propylcarbamoyloxy]-2-tetradecanoyloxypropyl] tetradecanoate (PEG2000-C-DMG); dierucoylphosphatidylcholine (DEPC); coronavirus disease 2019 (COVID-19).

**Table 2:** Examples of the U.S. Food and Drug Administration (FDA)-approved liposomal formulations and the different PK values obtained for adult versus pediatric populations. Reprinted from [161], copyright (2019), with permission from Elsevier.

Name	Lipids Used for Liposomes	Adult PK Parameters						Pediatric PK Parameters						Ratios of Pediatric Versus Adult PK Parameters				
		Dose	AUC_0–∞_ (ng/mL·h)	Cmax (ng/mL)	Tmax (h)	T1/2 (h)	CL (mL/min)	Dose	AUC_0–∞_ (ng/ml.hr)	Cmax (ng/mL)	Tmax (h)	T1/2 (h)	CL (mL/min)	AUC	Cmax	Tmax	T1/2	CL
Marqibo^®^ (Vincristine sulfate)	Sphingomyelin and cholesterol	2.25 mg/m^2^, i.v	14,566	1220	3.7	7.66	5.75	2.25 mg/m^2^, i.v	31,043	2150	1.12	10.7	1.2	2.13	1.76	0.3	1.39	0.2
SPI-77 (Cisplatin)	Soy PC, cholesterol and MPEG-DSPE.	200 mg/m^2^, i.v	13,850,680	82,538	N/A	103	0.29	200 mg/m^2^, i.v	24,004,000	2,414,000	N/A	78	0.15	1.73	29.24	N/A	0.75	0.51
DepoCyt^®^ (Cytarabine)	DOPC, DPPG, cholesterol	25 mg I/T	355,000	25,000	N/A	229	0.09	25 mg I/T	363,700	21,300	N/A	59.3	0.24	1.02	0.85	N/A	0.25	2.66
DaunoXome^®^ (Daunorubicin)	DSPC, cholesterol	80 mg/m^2^, i.v	10,330	400	N/A	0.77	233	80 mg/m^2^, i.v	108,206	900	N/A	12.63	7.6	10.47	2.25	N/A	16.4	0.03
AmBisome^®^ (Amphotericin B)	Soy PC, DSPG, alpha tocopherol, cholesterol	2 mg/kg i.v	288,000	22,900	N/A	6	0.16	5 mg/kg i.v	442,000	46,200	N/A	12.6	0.75	0.61	0.8	N/A	2.1	4.68

Abbreviations: area under the curve (AUC), the maximum observed concentration of the drug collected in bodily material from subjects in a clinical study (Cmax), clearance (CL), Intravenous (i.v), Intrathecal (I/T), time needed to reach the maximum concentration or time to Cmax (Tmax), half-life, is the time it takes for half the drug concentration to be eliminated (T_1/2_).

**Table 3:** Selected clinical trials that are currently recruiting or not yet recruiting using liposomal nanoformulations for pediatric interventions. Data were collected on 7 August 2023 from the ClinicalTrials.gov database, with inclusion criteria liposomes for children (birth to 17 years) in the recruiting or not-yet-recruiting status.

NCT Number	Phase	Study Status	Conditions	Interventions
Cancer				
NCT05739630	II and III	Recruiting	Acute Leukemia	Mitoxantrone liposome anti-thymocyte globulin
NCT04293562	III	Recruiting	Acute Myeloid Leukemia	Liposome-encapsulated daunorubicin-cytarabine, among others
NCT04606108	II	Recruiting	Soft Tissue Sarcoma	Camrelizumab in combination with liposome doxorubicin and ifosfamide
NCT05656248	II	Recruiting	Myeloid Neoplasm	Dual-drug liposomal encapsulation of cytarabine and daunorubicin (CPX-351)
NCT05620862	I	Recruiting	Lymphoma, Solid Tumors	Mitoxantrone hydrochloride liposome
NCT05457829	II	Not Yet Recruiting	Rhabdomyosarcoma, Child	Doxorubicin Hydrochloride Liposome+IrinotecanTemozolomide+Irinotecan+Vincristine
NCT04915612	I	Recruiting	Acute Myeloid Leukemia Arising from previous Myelodysplastic Syndrome	Gemtuzumab ozogamicin liposome-encapsulated daunorubicin-cytarabine
NCT05926492	II	Not Yet Recruiting	Osteosarcoma	Surufatinib plus chemotherapy, liposomal doxorubicin
NCT04996160	I	Recruiting	Acute Lymphoblastic Leukemia	Palbociclib, Dexamethasone, Bortezomib, Liposomal Doxorubicin
NCT04546620	II	Recruiting	Diffuse Large B Cell Lymphoma	R-CHOP, R-CHOP + acalabrutinib, Liposomal doxorubicin
NCT04199026	Early Phase I	Recruiting	Metastatic Sarcoma|Recurrent Sarcoma|Resectable Sarcoma	Liposomal doxorubicin, among others
NCT05518383	IV	Recruiting	Luymphoma	Liposomal doxorubicin, among others
NCT05315336	III	Not Yet Recruiting	Hemophagocytic Lymphohistiocytosis	Liposomal doxorubicin, etoposide, and methylprednisolone (L-DEP) and PD-1 antibody
NCT05561036	III	Recruiting	Desmoid Tumor	Liposome doxorubicin
NCT05675410	III	Recruiting	Lugano Classification Limited Stage Hodgkin Lymphoma AJCC v8	Liposomal doxorubicin, among others
NCT04791228	II	Recruiting	Solid tumors	Lyso-thermosensitive Liposomal Doxorubicin
NCT04984174		Recruiting	Pancreatic Cancer	Liposomal Irinotecan
NCT05576532	II	Recruiting	T-lymphoblastic Lymphoma	BCL2 Inhibitor plus IM2 regimen, Liposome mitoxantrone
NCT05711628	III	Not Yet Recruiting	Lymphoma	Pegylated Liposomal Doxorubicin Hydrochloride among others
NCT04589741	II	Recruiting	Soft Tissue Sarcoma	Toripalimab, Liposome adriamycin
NCT05210374	I	Recruiting	Relapsed Sarcomas	Liposomal doxorubicin, among others
Other pathologies				
NCT05730920	IV	Recruiting	Adolescent/Juvenile Idiopathic Scoliosis	Liposomal bupivacaine
NCT05714176	IV	Not Yet Recruiting	Chronic Kidney Disease (CKD)	Ferric Pyrophosphate Liposomal
NCT05468372	II	Recruiting	Mucormycosis; Pulmonary (Etiology)	Liposomal Amphotericin B
NCT04799236	III	Recruiting	Mucosal Leishmaniasis	Liposomal Amphotericin B, among others

**Table 4:** The use of chitosan in the development of user-friendly nanoformulations for pediatric applications.

Chitosan	Model Drug	Aim	Refs.
Chitosan with different molecular weights, degrees of deacetylation (DDA), and patterns of deacetylation	Prednisolone	To develop child-friendly solid dosage forms, e.g., oromucosal films and wafers	[173]
Low-molecular-weight chitosan (CS, 50–190 kDa, 75–85% deacetylation degree)	Cephalosporin	To formulate effective oral solutions of poorly soluble drugs suitable	[170]
Chitosan from *Portunus Sanguinolentus*	Dolutegravir	To adjust the dose	[174]
Medium molecular weight chitosan (190–310 KDa; 75–85% deacetylated)	Rufinamide	To reduce dose and dose frequency of Rufinamide by formulating Rufinamide-loaded chitosan nanoparticles suspended in a solution of a thermo-responsive polymer–tamarind seed xyloglucan for in situ gelling	[175]
Chitosan (90–95% deacetylation degree)	Cinnarizine	To develop chitosan microspheres for oral pediatric formulation with improved stability, organoleptic properties, and easier administration	[176]
Chitosan (approx. MW 296.6 kDa and deacetylation 82.83 ± 3.63%)	Didanosine	To make chitosan granules containing didanosine incorporated in chitosan microspheres, to facilitate handling and deglutition	[177]

**Table 5:** Completed clinical trials using chitosan. Data were collected on 9 August 2023 from the ClinicalTrials.gov database, with inclusion criteria “chitosan” for children (birth to 17 years) in the “completed” status.

NCT Number	Brief Summary	Conditions	Completion Date	Study Results
NCT00707486	The purpose of this study is to determine whether the HemCon Dental Dressing is effective in stopping bleeding during dental surgeries.	Tooth Extractions	1 July 2009	YES
NCT01597817	To evaluate the effect of a textile coated with chitosan in atopic dermatitis (AD) treatment as well as its impact on systemic inflammation and skin microbiome.	Atopic Dermatitis	1 December 2012	NO
NCT01950546	To evaluate the effectiveness of nanosilver fluoride for controlling the growth of *S. mutans* present in the dental plaque of children.	Dental Caries	1 January 2015	NO
NCT02789033	To assess the efficacy of the combination of isosorbide dinitrate spray and chitosan in diabetic foot ulcers.	Diabetic Foot Ulcers	1 August 2015	YES
NCT02668055	To evaluate the slow-release Tb4 collagen and chitosan porous sponge scaffolds skin substitutes and the effectiveness of clinical trials for the treatment of difficult-to-heal wounds and security.	Wounds	1 December 2015	NO
NCT05475444	PLGA nanoparticles coated with chitosan polymer were prepared and then incorporated in in situ gel to be injected into root canals of patients suffering from bacterial infection of their endodontics.	Bacterial Infections Oral	15 March 2020	NO
NCT04365270	To assess the antibacterial effect on carious dentine of glass ionomers when modified with chitosan and/or titanium dioxide nanoparticles versus the control group of modification with chlorhexidine when used in primary molars.	Caries	5 January 2021	NO
NCT03421717	Peri-implantitis is an inflammation in the mucosa surrounding an oral implant with loss of the supporting bone. The goals of peri-implantitis treatment are to resolve inflammation and arrest disease progression.	Periimplantitis|Peri-implant Mucositis	8 April 2021	NO
NCT04906291	To verify the caries-preventive efficacy of toothpaste containing biomimetic hydroxyapatite (H.A.) complex in children compared to traditional fluoridated toothpaste.	Caries	31 October 2021	NO
NCT04481945	To assess antimicrobial activity of nanosilver- and chitosan-inserted C sealer	Endodontic Disease	1 January 2022	NO
NCT04005872	The management of deep carious lesions.	Deep Caries	30 November 2022	NO

**Table 6:** Inorganic-based nanomedicines currently approved by the FDA [118].

Tradename	Formulation	Intervention	Approval Year
INFeD^®^	Iron Dextran Injection USP	Iron-deficient anemia	1992
DexFerrum^®^	Iron Dextran Injection USP	Iron-deficient anemia	1996
Ferrlecit^®^	Ferric gluconate (Rx)	Iron deficiency in chronic kidney disease	1999
Venofer^®^	Iron sucrose injection	Iron deficiency in chronic kidney disease	2000
Feraheme^®^	Ferumoxytol injection	Iron deficiency in chronic kidney disease	2009
Injectafer^®^	Ferric carboxymaltose injection	Iron-deficient anemia	2013

**Table 7:** EMA (EMA/CAT/50775/2023)- and FDA [219]-approved advanced therapies medicinal products (ATMPs).

Name	Type of ATMP	Indication	Approval
		Cell therapy medicinal product.	
Allocord Clevecord Ducord Hemacord	Cord blood HPC	Indicated for use in unrelated-donor hematopoietic progenitor stem cell transplantation procedures.	FDA
Ebvallo	Allogeneic T-cell immunotherapy	To treat adults and children from 2 years of age who, after receiving an organ or a bone marrow-transplantation, develop a blood cancer called Epstein–Barr virus positive post-transplant lymphoproliferative disease (EBV + PTLD).	EMA
Omisirge	Cord blood nicotinamide modified allogeneic hematopoietic progenitor cells	Used in adults and pediatric patients 12 years and older with hematologic malignancies who have planned umbilical cord blood transplantation following myeloablative conditioning to reduce the time to neutrophil recovery and the incidence of infection.	FDA
		Gene therapy medicinal product.	
Elevidys	Non-replicating, recombinant adeno-associated virus for delivery of *Micro-dystrophine* gene	To treat ambulatory pediatric patients aged 4–5 years with Duchenne muscular dystrophy (DMD) with a confirmed mutation in the micro-dystrophine gene.	FDA
Luxturna	Adeno-associated viral vector serotype 2 for delivery of *RPE65* gene	To treat adult and pediatric patients with confirmed biallelic *RPE65* mutation-associated retinal dystrophy.	FDA EMA
Skysona	Autologous CD34+ enriched H*SCs* transduced ex vivo with lentiviral vector encoding ABCD1 complementary deoxyribonucleic acid (cDNA) for human adrenoleukodystrophy protein	To slow the progression of neurologic dysfunction in male patients 4–17 years of age with early, active cerebral adrenoleukodystrophy with no available sibling hematopoietic stem cell donor.	FDA No longer authorized by EMA
Vyjuvek	Herpes-simplex virus type 1 vector for delivery of *COL7A1* gene	To treat wounds in patients with 6 months of age and older with dystrophic epidermolysis bullosa with mutation(s) in the collagen type VII alpha 1 chain (*COL7A1*) gene.	FDA
Zynteglo	Autologous CD34+ enriched hematopoietic stem cells transduced ex vivo with lentiglobin BB305 lentiviral vector encoding *beta-A-T87Q-globin* gene	Treatment of adult and pediatric patients with ß-thalassemia who require regular red blood cell (RBC) transfusions.	FDA No longer authorized by EMA
Zolgensma	Adeno-associated viral vector serotype 9 encoding the *Survival Motor Neuron 1* gene	Treatment of Spinal Muscular Atrophy (Type I) for pediatric patients under 2 years of age.	FDA EMA
Strimvelis	Autologous CD34+ enriched HSC transduced with retroviral vector that encodes for the human ADA cDNA sequence	To treat patients with severe combined immunodeficiency due to adenosine deaminase deficiency (ADA-SCID).	EMA
Kymriah	Genetically modified (chimeric antigen receptor) autologous T cell immunotherapy	To treat children or young adults (up to 25 years old) with Acute Lymphoblastic Leukemia.	EMA
Libmeldy	Autologous hematopoietic stem and progenitor cell transfected ex vivo with lentiviral vector encoding the human *Arylsulfatase A* gene	To treat children with metachromatic leukodystrophy.	EMA
Upstaza	Adeno-associated viral vector encoding *L-amino Acid Decarboxylase* gene	To treat adults and children aged 18 months and older with severe aromatic L-amino acid decarboxylase deficiency with a genetically confirmed diagnosis.	EMA
		Tissue engineered product	
Rethymic	Allogeneic processed thymus tissue	Immune reconstitution in pediatric patients with congenital athymia.	FDA
Spherox	Chondrocyte spheroids	To repair defects to the cartilage in the knee in patients who are experiencing symptoms (such as pain and problems moving the knee) in adults and adolescents.	EMA
