# Correction: Domingues et al. Pediatric Drug Development: Reviewing Challenges and Opportunities by Tracking Innovative Therapies. *Pharmaceutics* 2023, *15*, 2431

**DOI:** 10.3390/pharmaceutics17080994

**Published:** 2025-07-31

**Authors:** Cátia Domingues, Ivana Jarak, Francisco Veiga, Marília Dourado, Ana Figueiras

**Affiliations:** 1 Univ Coimbra, Laboratory of Drug Development and Technologies, Faculty of Pharmacy, 3000-548 Coimbra, Portugal; cdomingues@ff.uc.pt (C.D.); jarak.ivana@gmail.com (I.J.); fveiga@ci.uc.pt (F.V.); 2 LAQV-REQUIMTE, Laboratory of Drug Development and Technologies, Faculty of Pharmacy, University of Coimbra, 3000-548 Coimbra, Portugal; 3 Univ Coimbra, Institute for Clinical and Biomedical Research (iCBR) Area of Environment Genetics and Oncobiology (CIMAGO), Faculty of Medicine, 3000-548 Coimbra, Portugal; mdourado@fmed.uc.pt; 4 Institute for Health Research and Innovation (i3s), University of Porto, 4200-135 Porto, Portugal; 5 Univ Coimbra, Center for Health Studies and Research of the University of Coimbra (CEISUC), Faculty of Medicine, 3000-548 Coimbra, Portugal; 6 Univ Coimbra, Center for Studies and Development of Continuous and Palliative Care (CEDCCP), Faculty of Medicine, 3000-548 Coimbra, Portugal

## Text Correction

There were some aspects in the original publication that need to be corrected [1]. A lapse in the wording affected the description of the transdermal and dermal routes of administration. Therefore, to avoid misleading and to improve the readability, a correction has been made to Section 3.2.4 Administration Route and Pharmaceutical Dosage Forms in Pediatrics, Paragraph 7, as outlined below:

The sentence “Dealgaro-Charro and Guy [95] have previously reviewed the importance of transdermal drug delivery in children” was added later in the same Section 3.2.4 as follows: “Their relevance in children has been recently reviewed by Delgado-Charro & Guy [95]”. In the description of the outcomes in “Section 3.2.4: Administration Route and Pharmaceutical Dosage Forms in Pediatrics”, a minor modification to the “Transdermal dosage forms (…)” was requested. Therefore, the statement has been modified as follows: “Pharmaceutical dosage forms intended for dermal (or cutaneous) administration are tailored to promote a local effect. They can encompass liquid preparations, such as lotions or shampoos; semi-solid preparations, like ointments or creams; as well as solid preparations, e.g., powders [12]. Accidental systemic absorption through the dermis of APIs could have a dramatic impact on preterm neonates, as the stratum corneum is deficient, and in children as the volume of distribution per unit of skin area is lower [12].”

In the same Section 3.2.4, another modification was requested and the text was changed as follows: “On the other hand, transdermal administration, for example using patches, is intended for systemic delivery of APIs that are capable of diffusion through the stratum corneum [12]. Their relevance in children has been recently reviewed by Delgado-Charro & Guy [95]. Depending on the dosage form, the assessment of excipient safety is of the utmost importance (see Section 3.2.5). Although not appropriate for all drugs, various transdermal patches containing active drug ingredients such as fentanyl, clonidine, or scopolamine have been exploited for pediatric applications [95].”

Moreover, the abbreviations for propylene glycol and polyethylene glycol were not properly stated. A correction has been made to Section 3.2.5. Excipients, Paragraphs 2 and 4: 

Paragraph 2: “Of relevance are propylene glycol (PG) and ethanol, which cannot be metabolized in the same manner as in adults due to the immaturity of the organs, particularly in young children [114].”

Paragraph 4: “The evaluation of certain excipients have been prioritized, such as benzalkonium chloride, benzoic acid and benzoates, benzylalcohol, cyclodextrins, ethanol, and PG, as some reports had revealed that they cause more damage and side effects in the pediatric population [14,116].”

Paragraph 4: “On the other hand, Polyethylene glycol (PEG) has been extensively utilized in pharmaceutical formulations, not only as an API (e.g., PEG 3350 or PEG 4000 in colonoscopy preparations or laxative solutions), but more commonly as a multifunctional excipient due to its broad range of physicochemical properties and applications [117].”

The authors state that the scientific conclusions are unaffected. This correction was approved by the Academic Editor. The original publication has also been updated.
